# A Herbal‐Piezoelectric Heterojunction Strategy to Potentiate Bacterial Cuproptosis‐Like Death and Remodel the Inflammatory Microenvironment in Infection‐Associated Implant Osteomyelitis

**DOI:** 10.1002/advs.202506362

**Published:** 2025-08-28

**Authors:** Guannan Zhang, Zehao Li, Ying Lu, Jianbo Song, Xingyu Liang, Peide Han, Xiaohong Yao, Yongqiang Yang, Xiangyu Zhang

**Affiliations:** ^1^ Shanxi Bethune Hospital Shanxi Academy of Medical Sciences Third Hospital of Shanxi Medical University Tongji Shanxi Hospital Taiyuan 030032 China; ^2^ Shanxi Provincial Key Laboratory for Translational Nuclear Medicine and Precision Protection Taiyuan 030006 China; ^3^ Shanxi Key Laboratory of Biomedical Metal Materials College of Materials Science and Engineering Taiyuan University of Technology Taiyuan 030024 China; ^4^ The First Hospital of Shanxi Medical University Taiyuan 030001 China; ^5^ College of Materials Science and Engineering Taiyuan University of Technology Taiyuan 030024 China; ^6^ College of Biomedical Engineering Taiyuan University of Technology Taiyuan 030024 China; ^7^ National Graphene Products Quality Inspection and Testing Center (Jiangsu) Special Equipment Safety Supervision Inspection Institute of Jiangsu Province Wuxi 214174 China

**Keywords:** acoustoelectric therapy, bacterial cuproptosis‐like death, herbal, immunoregulation, osseointegration

## Abstract

For infection‐associated implant osteomyelitis (IAOM) caused by *Staphylococcus aureus*, copper‐induced bacterial death holds great potential. However, inducing bacterial cuproptosis‐like death using low concentrations of copper (Cu) ions remains a challenge. Therefore, a “herbal‐piezoelectric heterojunction” (Herbal‐Piezo‐HJ) is designed by integrating berberine (Ber) with Cu ion‐doped Strontium titanate (SrTiO_3_, STO) on a Ti substrate (STO‐Cu@Ber). Ber and acoustoelectric therapy (SPT) synergistically enhance the efficacy of low‐concentration Cu ions in inducing bacterial cuproptosis‐like death. The formation of inorganic/organic heterojunctions significantly enhances piezoelectric performance, generating marked electronic asymmetry at the interface under ultrasonic (US) irradiation and producing a large amount of reactive oxygen species (ROS). ROS and Ber together damage bacterial cell walls and membranes, promoting Cu ion uptake into bacterial cells. Consequently, the bacterial glycolytic respiratory mode is altered, the tricarboxylic acid cycle is significantly inhibited, and a large amount of nutrients are leaked, resulting in bacterial cuproptosis‐like death. Additionally, Herbal‐Piezo‐HJ downregulates arachidonic acid metabolites, activates the cyclic adenosine monophosphate (cAMP) signaling pathway to resolve the inflammatory response after infection clearance, and promotes bone integration. Overall, the combination of herbal therapy and SPT to enhance bacterial cuproptosis‐like death offers a promising strategy for eradicating IAOM.

## Graphical abstract

A titanium implant coating integrating berberine and copper‐doped strontium titanate leverages ultrasound to compromise bacterial membranes and induce cuproptosis‐like death. This approach resolves *S. aureus* infections in osteomyelitis models while suppressing inflammation and promoting osseointegration, demonstrating a dual‐functional strategy against implant‐associated complications.

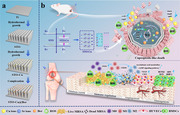

## 1 Introduction

Implant‐associated osteomyelitis (IAOM), induced by bacterial infection, is recognized as a prevalent and severe complication in orthopedic surgery.^[^ ^1^ ^]^ While various microorganisms have been linked to bone infections, *Staphylococcus aureus* (*S. aureus*) remains the most common and severe causative bacterium.^[^ ^1a^ ^]^ Currently, the clinical management of IAOM still relies on high‐dose antibiotics.^[^ ^2^ ^]^ However, antibiotics are difficult to transport to deep infection sites, and their overuse can affect patient's immune system and potentially lead to the emergence of multidrug‐resistant bacteria.^[^ ^3^ ^]^ Notably, up to 50% of *S. aureus* osteomyelitis cases are attributed to methicillin‐resistant *Staphylococcus aureus* (MRSA) strains.^[^ ^1b^ ^]^

Copper (Cu) is an ancient and classic broad‐spectrum antibacterial metal element.^[^ ^4^ ^]^ In 2008, the United States Environmental Protection Agency officially recognized copper and its alloys as the first effective metal antibacterial agents.^[^ ^5^ ^]^ Recent studies have demonstrated that Cu accumulation in bacterial biofilms induces cuproptosis‐like death by interacting with components of the tricarboxylic acid (TCA) cycle.^[^ ^6^ ^]^ However, the antibacterial activity of Cu ions shows a dose‐dependent relationship.^[^ ^7^ ^]^ The minimum inhibitory concentration of Cu ions against bacteria is 5 ppm, which is very close to the cytotoxic concentration (7.8 ppm).^[^ ^5^, ^8^ ^]^ Recent studies demonstrate that some strategies achieve antibacterial effects with reduced cytotoxicity by lowering doses of Cu ions that trigger cuproptosis‐like death in bacteria. For instance, bacterial cuproptosis‐like death can be triggered by reactive oxygen species (ROS) and hyperthermia through dual disruption of the cell wall/membrane and accelerated Cu ion entry.^[^ ^8^, ^9^ ^]^ Additionally, improving the hypoxic microenvironment and altering the glycolytic respiratory mode of bacteria can also increase their sensitivity to cuproptosis‐like death.^[^ ^4^, ^10^ ^]^

An increasing number of researchers are now using Chinese herbal medicines as biofunctional materials to treat cancer or bacterial infections.^[^ ^11^ ^]^ The sustainable nature and good biocompatibility of natural products contribute to their value as a source of drug molecules. Yu et al. prepared nanoparticles that enhance cuproptosis‐like death in lung cancer by depleting glutathione (GSH) and enhancing immune responses using an extract of the traditional Chinese herb Tripterygium wilfordii (triptolide).^[^ ^12^ ^]^ However, research on enhancing cuproptosis‐like death in bacterial cells using Chinese herbal medicine has not yet been reported. Active ingredients in certain Chinese herbal medicines may interact with components of bacterial cell membranes.^[^ ^13^ ^]^ For instance, some flavonoids are lipophilic and can integrate into the phospholipid bilayer of membranes.^[^ ^14^ ^]^ Extracellular herbal components may disrupt membrane integrity, thereby promoting intracellular Cu ion accumulation in bacterial cells.^[^ ^12^ ^]^ Certain components of Chinese herbal medicines may also disrupt the function of bacterial Cu transporters by altering their conformation or inhibiting their ability to bind Cu ions,^[^ ^14^ ^]^ thereby hindering Cu ion transport within bacteria. Therefore, we propose that specific Chinese herbal medicines may potentiate bacterial cuproptosis‐like death. Furthermore, to reduce the required concentration of Cu ions and more efficiently induce cuproptosis‐like death, it may be necessary to combine other approaches that enhance this effect, such as ROS generation.

In recent years, the unique mechano‐electrical conversion properties of piezoelectric catalysis have garnered significant attention.^[^ ^15^ ^]^ Acoustic catalysts based on piezoelectric semiconductor materials have shown promising potential in ultrasound‐mediated therapy, referred to as acoustoelectric therapy (SPT).^[^ ^16^ ^]^ Ultrasound (US) can induce mechanical stress in piezoelectric materials and generate a piezoelectric field.^[^ ^16b^ ^]^ This process regulates charge carrier migration, induces band bending, and facilitates continuous separation of electrons and holes toward the material surface, promoting redox reactions.^[^ ^17^ ^]^ Some piezoelectric materials (zinc oxide, black phosphorus, barium titanate, etc.) have been used in SPT.^[^ ^18^ ^]^ However, there is a need for piezoelectric materials that exhibit strong binding affinity to titanium (Ti) substrates while also possessing biocompatibility and osteogenic properties. Strontium titanate (SrTiO_3_, STO) is a typical piezoelectric material with excellent biocompatibility and favorable mechanical and dielectric properties.^[^ ^19^ ^]^ STO can be synthesized from Ti‐based implants and tightly bound to the matrix.^[^ ^20^ ^]^ Moreover, strontium is a bioactive, osteophilic element that stimulates osteoblast proliferation and differentiation, enhances bone formation, and inhibits bone resorption.^[^ ^20^ ^]^ Thus, STO represents an ideal choice for fabricating piezoacoustic‐sensitive coatings. However, the microenvironment of osteomyelitis, characterized by hypoxia, weak acidity, and elevated hydrogen peroxide (H_2_O_2_) levels, can significantly impair therapeutic efficacy.^[^ ^21^ ^]^ This issue can currently be addressed through the following approaches: 1) augmenting catalytic activity to enhance ROS production, and 2) improving the hypoxic microenvironment.^[^ ^22^ ^]^

Strategies that utilize defect engineering‐guided ion doping can significantly enhance piezoelectric catalytic efficiency.^[^ ^23^ ^]^ Cu ion doping not only improves SPT efficiency but also enables chemodynamic therapy (CDT) via valence transitions between Cu⁺ and Cu^2^⁺.^[^ ^9b^ ^]^ This process facilitates oxygen production through Fenton‐like reactions with highly expressed H_2_O_2_ in the infected microenvironment, thereby improving hypoxia.^[^ ^24^ ^]^ Additionally, modulating the energy band structure by constructing heterojunctions is an effective strategy for enhancing SPT, as it improves the separation and transmission efficiency of electrons and holes.^[^ ^25^ ^]^ Among various types of heterojunctions, variations in material density at the interface of organic–inorganic heterojunctions create an acoustic impedance gradient.^[^ ^26^ ^]^ This gradient facilitates the generation of US echoes and enhances US absorption. Wu et al. constructed an organic–inorganic hybrid Cur/CuS heterojunction to improve electron–hole pair separation via photoacoustic interface engineering.^[^ ^27^ ^]^ Berberine (Ber) is an herbal sonosensitizer known for its antibacterial, anti‐inflammatory, and immunomodulatory properties.^[^ ^28^ ^]^ Therefore, Ber‐based heterojunctions may potentially amplify bacterial cuproptosis‐like death via dual pathways: intrinsic herbal bioactivity and enhanced sonodynamic efficiency.

**Scheme 1:**
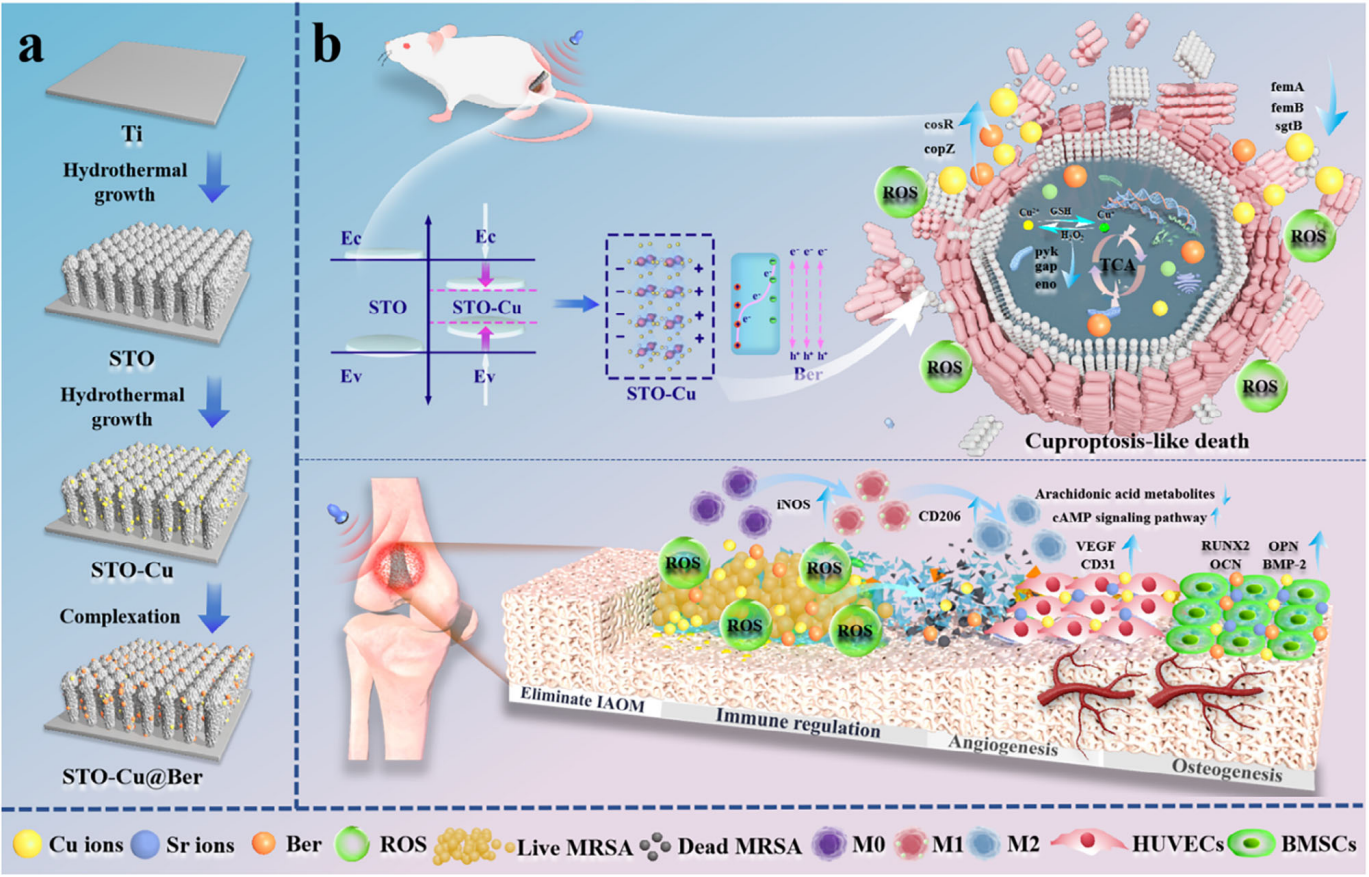
Schematic illustration of STO‐Cu@Ber for the removal of IAOM and promotion of bone regeneration.

Hence, we fabricated a “herbal‐piezoelectric heterojunction” (Herbal‐Piezo‐HJ) by integrating Ber with Cu ion‐doped STO on a Ti substrate (STO‐Cu@Ber) (Scheme 1). The construction of heterojunctions can potentially improve piezoelectricity by inducing electron imbalance at inorganic/organic interfaces, thereby narrowing the excitation bandgap energy. Under US irradiation, the Herbal‐Piezo‐HJ generates a substantial quantity of ROS. The combination of ROS and Ber synergistically disrupts the bacterial cell wall and membrane, enhances membrane permeability, facilitates the uptake of Cu ions and Ber by bacteria, and shows excellent antibacterial activity against MRSA. Transcriptomic analysis shows that the TCA cycle of MRSA is significantly inhibited, leading to the accumulation of lipoylated proteins and loss of iron–sulfur cluster proteins, ultimately resulting in cuproptosis‐like death. Metabolomics analysis indicates that downregulation of arachidonic acid metabolites and upregulation of the cyclic adenosine monophosphate (cAMP) signaling pathway can effectively alleviate the inflammatory response following infection clearance. Furthermore, Herbal‐Piezo‐HJ promotes angiogenesis post‐infection, upregulates osteogenic factors, and comprehensively enhances osseointegration. The ability of Herbal‐Piezo‐HJ to eliminate bacteria through ultrasound‐mediated treatment demonstrates considerable clinical potential for the management of IAOM.

## 2 Results and Discussion

### 2.1 Synthesis and Characterization

**Figure 1:**
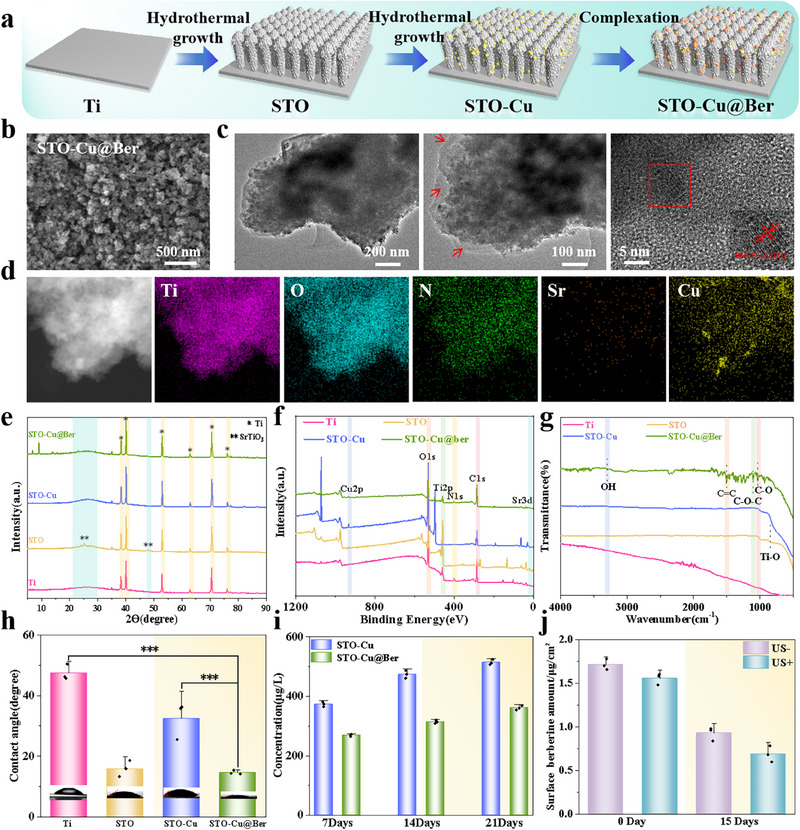
a) Schematic illustration of the preparation process for STO‐Cu@Ber; b) Field‐emission scanning electron microscopy (FE‐SEM) and c) TEM images of STO‐Cu@Ber; d) EDS mapping of STO‐Cu@Ber; e) XRD patterns, f) XPS survey spectra and g) FTIR spectra of Ti, STO, STO‐Cu and STO‐Cu@Ber; h) Water contact angles of Ti, STO, STO‐Cu and STO‐Cu@Ber (n = 3); i) Cu ion release profiles in STO‐Cu and STO‐Cu@Ber at days 7, 14, and 21 (n = 3); j) Ber content in STO‐Cu@Ber surface (n = 3). Error bars represent ± SD. ***p < 0.001 by Student's *t*‐test.

The preparation process of STO‐Cu@Ber is shown in **Figure** **1**a. Titanate nanoarrays were initially synthesized following our previously established method.^[^ ^29^ ^]^ STO was then produced via hydrothermal treatment using strontium hydroxide, followed by doping with Cu ions through a secondary hydrothermal treatment to yield STO‐Cu. Subsequently, the complexing effect of Ber on metal ions was employed to further modify STO‐Cu with Ber, resulting in STO‐Cu@Ber. As shown in Figures S1 and S2 (Supporting Information), a nanostructure resembling a short, rough baseball bat formed on the Ti plate following the alkaline thermal reaction, with a coating thickness of ≈650 nm. Cu ion doping and Ber modification did not alter the fundamental morphology, but the surface roughness decreased from 64.2 nm to 45.8 and 36.2 nm, respectively (Figures S1 and S3, Supporting Information). The TEM image shows that the lattice spacing of the synthesized STO is 0.254 nm, corresponding to the (110) crystal plane (Figure S2, Supporting Information). Notably, Cu ion doping did not alter the crystalline structure, and an organic layer was observed on the surface (indicated by the red arrows), confirming the modification with Ber (Figure 1c). Energy‐dispersive X‐ray spectroscopy (EDS) (Figure 1d) indicates that the elements Ti, O, N, Si, and Cu are uniformly distributed across the surface of STO‐Cu@Ber. Inductively coupled plasma optical emission spectrometry (ICP‐OES) revealed that the elemental contents of Sr and Cu in STO‐Cu@Ber were 110.425 and 6.7 mg kg^−1^, respectively. The diffraction peak at 24.4° in the X‐ray diffraction (XRD) spectrum further confirms that STO with the (110) crystal plane was successfully synthesized. Cu doping and Ber modification did not significantly affect the phase structure of STO. However, STO‐Cu exhibits a slight distortion in the diffraction peak between 25° and 30°, likely due to Cu ion doping.^[^ ^30^ ^]^

X‐ray photoelectron spectroscopy (XPS) revealed that C 1s, O 1s, and Ti 2p peaks were present in all samples, while Sr 3d, Cu 2p, and N 1s peaks were observed specifically in STO, STO‐Cu, and STO‐Cu@Ber, respectively (Figure 1f). The high‐resolution C 1s XPS spectrum of STO‐Cu@Ber exhibits characteristic peaks corresponding to C═O, C─O, C─N, C─C, and C═C at binding energies of 287.7, 286.3, 285.4, 284.6, and 284.1 eV, respectively (Figure S5, Supporting Information).^[^ ^30^ ^]^ In addition, fourier transform infrared spectroscopy (FTIR) results indicate the presence of functional groups such as OH, C═C, C─O─C, and C─O bonds (Figure 1g), confirming the successful modification with Ber. This confirms the successful modification of Ber. The contact angles for Ti, STO, STO‐Cu, and STO‐Cu@Ber were 47.4°, 15.1°, 32.3°, and 14.8°, respectively (Figure 1h). These variations in hydrophilicity likely originate from surface morphological and chemical modifications.

Figure 1i illustrates the release concentrations of Cu ions from both STO‐Cu and STO‐Cu@Ber over various time intervals. After soaking for 7, 14, and 21 days, the release concentration of Cu ions in both materials gradually increased. However, the release amount of Cu ions in STO‐Cu@Ber remained relatively low. This phenomenon may be attributed to the surface modification of STO‐Cu@Ber by Ber, which appears to impede Cu ion release. Figure 1j shows the amount of Ber retained on the surface of STO‐Cu@Ber with or without US irradiation. On day 0, the amounts were 1.71 and 1.56 µg cm^−^^2^, respectively. By day 15, these values decreased to 0.96 and 0.62 µg cm^−^^2^, respectively. The results suggest a sustained Ber release profile, while US irradiation appears to enhance this process. This observation is consistent with the Ber release curve presented in Figure S6 (Supporting Information).

### 2.2 In Vitro Sonodynamic Performance and Density Functional Theory (DFT) Calculation

**Figure 2:**
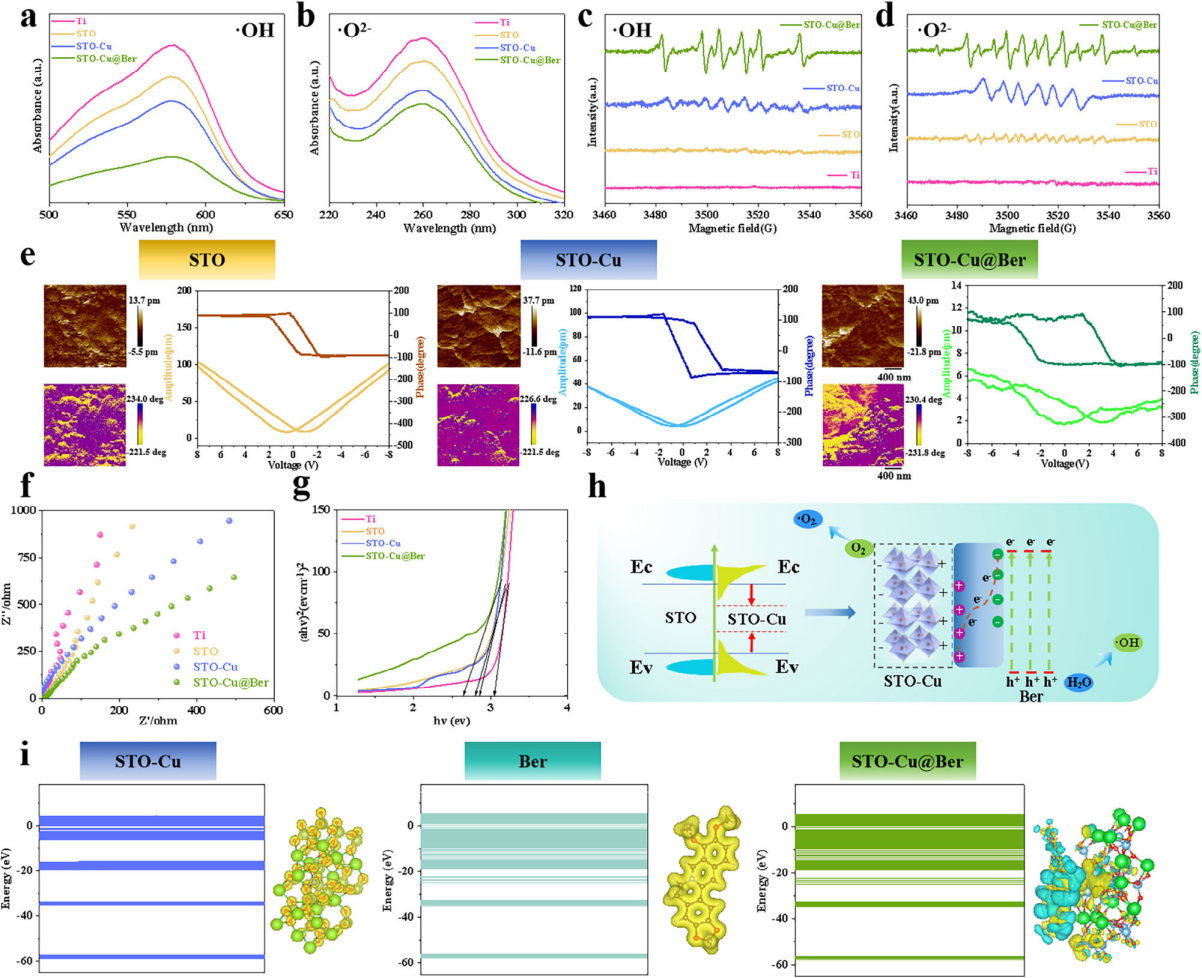
a) MV and b) NBT degradation of Ti, STO, STO‐Cu and STO‐Cu@Ber; c) EPR spectrum of ·OH and d) ·O^2−^; e) Amplitude‐voltage curves and phase hysteresis loops of STO, STO‐Cu and STO‐Cu@Ber; f) EIS test and g) UV–vis DRS curves of Ti, STO, STO‐Cu and STO‐Cu@Ber; h) Schematic illustration of the ROS generation mechanism of STO‐Cu@Ber under US irradiation; i) The energy band structures and the electron cloud distribution of STO‐Cu, Ber and STO‐Cu@Ber.

The sonodynamic activity was evaluated via hydroxyl radical (·OH) and superoxide anion radical (·O_2−_) detection during US irradiation using methyl violet (MV) and nitroblue tetrazolium (NBT) degradation assays.^[^ ^31^ ^]^ Compared to Ti, the intensity of the absorption peak of STO is slightly diminished. Following Cu ion doping, the intensity of the absorption peak of STO‐Cu is further reduced. Notably, the absorption peak of STO‐Cu@Ber, after modification with Ber, exhibits the most significant decrease (**Figure** **2**a,b). Figure S7 (Supporting Information) illustrates that the ROS yield of STO‐Cu@Ber is positively correlated with the duration of irradiation. Furthermore, electron spin resonance (EPR) spectroscopy confirms that STO‐Cu@Ber demonstrates optimal sonodynamic properties following Cu ion doping and modification with Ber (Figure 2c,d).

The band tilt theory proposes electron transitions from valence to conduction bands in sonosensitizers during sonopiezocatalysis, with valence band hole formation under US irradiation.^[^ ^32^ ^]^ Concurrently, US waves induce mechanical deformation of the sonosensitizer, triggering the piezoelectric effect and generating a built‐in electric field within the material.^[^ ^32^ ^]^ This built‐in electric field drives the opposite‐direction transport of electrons and holes, enhancing charge carrier separation and migration efficienc. The piezoelectric properties of STO, STO‐Cu, and STO‐Cu@Ber were characterized using piezoresponse force microscopy (PFM). The characteristic piezoelectric butterfly loops and 180° phase hysteresis illustrated in Figure 2e confirm that STO, STO‐Cu, and STO‐Cu@Ber exhibit strong piezoelectric properties. Notably, compared to STO, both Cu ion doping and Ber modification resulted in an increase in the maximum amplitude and the phase transition voltage difference for STO‐Cu and STO‐Cu@Ber, underscoring their enhanced piezoelectricity. This enhancement in piezoelectric response sensitivity is primarily attributed to oxygen vacancies (*O*_v_) and lattice distortion induced by Cu ion doping, which increases crystal asymmetry (Figure S8, Supporting Information). In addition, grafting Ber may alter the energy band structure and accelerate charge transfer, further enhancing piezoelectric properties. We assessed how ion doping and heterostructures enhance piezocatalysis by influencing charge separation and band structure. Electrochemical analysis evaluated carrier behavior, with US‐induced current densities shown in Figure S9 (Supporting Information). During US application, the current generated by STO, STO‐Cu, and STO‐Cu@Ber remains stable, with a sequential increase in intensity. In STO‐Cu, *O*_v_ act as electron traps, suppressing surface carrier recombination and enhancing electron utilization efficiency. The STO‐Cu@Ber heterojunction promotes carrier separation and migration, thereby generating significantly stronger transient acoustic currents. Electrochemical impedance spectroscopy (EIS) was used to assess charge transfer characteristics. A reduction in the diameter of the Nyquist circle indicates enhanced carrier separation and conversion efficiency (Figure 2f). The arc radius of the Nyquist curves for Ti, STO, STO‐Cu, and STO‐Cu@Ber shows a gradual decrease, with STO‐Cu@Ber demonstrating the highest interfacial charge transfer efficiency, consistent with the photocurrent test results. These findings suggest that the introduction of *O*_v_ and the formation of heterojunctions accelerate carrier transfer rates and improve electron–hole separation. In addition, the bandgaps of Ti, STO, STO‐Cu, and STO‐Cu@Ber, measured by UV–vis DRS, also show a decreasing trend (Figure 2g).

The energy band structure and interfacial charge transfer pathways of STO‐Cu, Ber, and STO‐Cu@Ber were further evaluated through DFT calculations. As shown in Figure 2i, the theoretical bandgap value of STO‐Cu@Ber was reduced compared to that of STO‐Cu, consistent with the measured trend. Differential charge density calculations were used to assess the effect of charges at the STO‐Cu@Ber interface. The delocalization effect induces uniform electron distribution across STO‐Cu and Ber, forming a stabilized electronic configuration. Charge density variations at the interface confirm electron redistribution upon their combination. Heterojunction formation disrupts Ber's conjugated structure, triggering electron transfer from Ber to the STO‐Cu surface, with altered charge distributions localized at the interface. This interfacial charge imbalance drives efficient electron–hole separation. Oxygen adsorption near the interface accelerates electron transfer to oxygen species, inducing rapid destabilization. The construction of the Herbal‐Piezo‐HJ reduces the interfacial bandgap, induces charge imbalance, and enhances the efficiency of SPT.

### 2.3 Antibacterial Effects In Vitro

**Figure 3:**
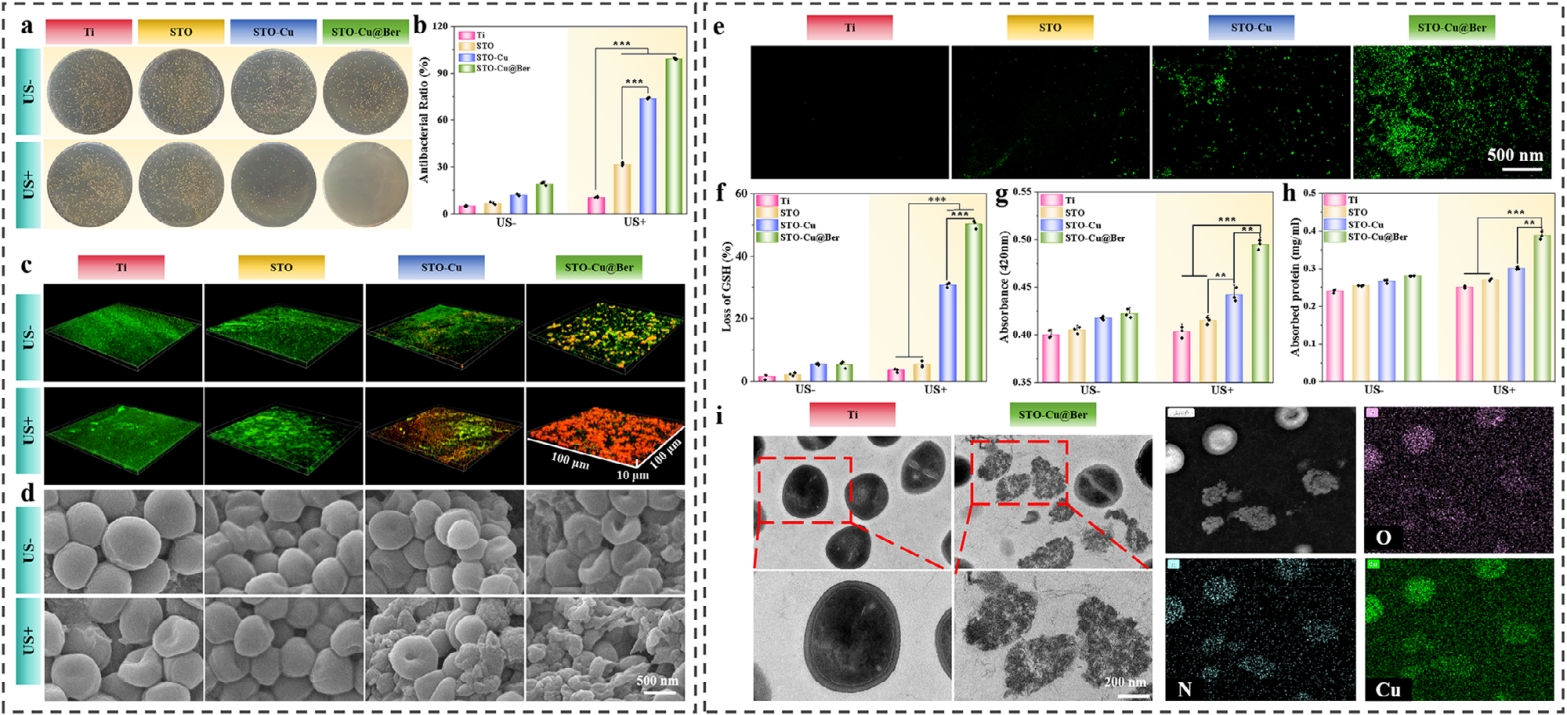
a) Photographs of bacterial colonies, b) Antibacterial rates (n = 3), c) Bacterial viability assessment via live/dead fluorescence, and d) SEM images of bacterial on Ti, STO, STO‐Cu and STO‐Cu@Ber with or without US irradiation; e) Fluorescent staining images of ROS in bacteria under US irradiation; f) GSH degradation (n = 3), g) ONPG substrate conversion (n = 3) and h) Membrane integrity evaluation through protein leakage (n = 3); i) Ultrastructural analysis by TEM with elemental mapping of bacterial on Ti and STO‐Cu@Ber after US irradiation. Error bars represent ± SD. **p < 0.01, ***p < 0.001 by Student's *t*‐test.

Given the favorable sonodynamic effect of STO‐Cu@Ber, we investigated whether SPT and Ber could further enhance the induction of cuproptosis‐like death in *S. aureus* cells. The bacterial colony plate results indicate that, without US irradiation, the antibacterial effect of each sample group is not significant. The antibacterial properties of Cu ions and Ber contributed to the observed antibacterial rates of STO‐Cu and STO‐Cu@Ber, which were 12.1% and 19.4%, respectively (**Figure** **3**a,b). Furthermore, the antibacterial rates for equivalent amounts of Cu ions and Ber immobilized on the sample surface were only 8.5% and 6.8%, respectively (Figure S10, Supporting Information). Ti and STO exhibited minimal antibacterial effects under US irradiation. In contrast, the antibacterial rates of STO‐Cu and STO‐Cu@Ber reached 73.8% and 99.2%, respectively. The antibacterial efficacy of STO‐Cu@Ber under US irradiation was further evaluated using the live/dead fluorescence staining method (Figure 3c). As anticipated, only STO‐Cu@Ber demonstrated the ability to rapidly eliminate *S. aureus* biofilm under US irradiation. The morphology of bacteria was observed by SEM (Figure 3d). In the absence of US irradiation, bacteria on Ti, STO, and STO‐Cu surfaces exhibited a spherical shape with intact cell membranes, whereas bacteria on the STO‐Cu@Ber surface displayed a concave shape. Under US irradiation, bacteria on Ti and STO surfaces maintained a typical spherical shape. In contrast, bacteria on the STO‐Cu surface appeared dented and shrunken, while those on the STO‐Cu@Ber surface showed severe shrinkage and distortion.

Given the substantial generation of ROS during the SPT process, fluorescent staining was employed to assess ROS levels in bacterial cells under US irradiation. As shown in Figure 3e, minimal green fluorescence was observed on the STO‐Cu surface, whereas a significant amount was detected on the STO‐Cu@Ber surface. This indicates that STO‐Cu@Ber can generate high levels of ROS that are lethal to bacteria under US irradiation. In bacteria, GSH is converted to glutathione disulfide (GSSG) in response to oxidative stress.^[^ ^30^ ^]^ This conversion serves as a critical metric for quantifying intracellular oxidative stress levels. Both ROS and Ber can damage bacterial cell membranes, thereby accelerating the entry of Cu ions into bacterial cells. To investigate this, we assessed the impact of STO‐Cu@Ber on bacterial membrane integrity under US irradiation. The permeability of the bacterial membrane was evaluated using o‐nitrophenyl‐β‐D‐galactopyranoside (ONPG). As shown in Figure 3g, only bacteria on the STO‐Cu@Ber surface exhibited significant β‐galactosidase release under US irradiation. Furthermore, membrane damage led to a marked increase in protein leakage from bacterial cells on the STO‐Cu@Ber surface. The morphology of the bacterial membrane was further examined by TEM. Notably, bacterial membranes on the STO‐Cu@Ber surface showed significant rupture following US irradiation (Figure 3i). Additionally, elemental mapping clearly indicated the infiltration of Cu ions into the interior of bacterial cells, with the atomic fraction of Cu reaching as high as 7.8% (Table S1, Supporting Information), far exceeding that of untreated bacteria.

### 2.4 Transcriptome Sequencing Analysis of the Bacterial Cuproptosis‐Like Death Mechanism

**Figure 4:**
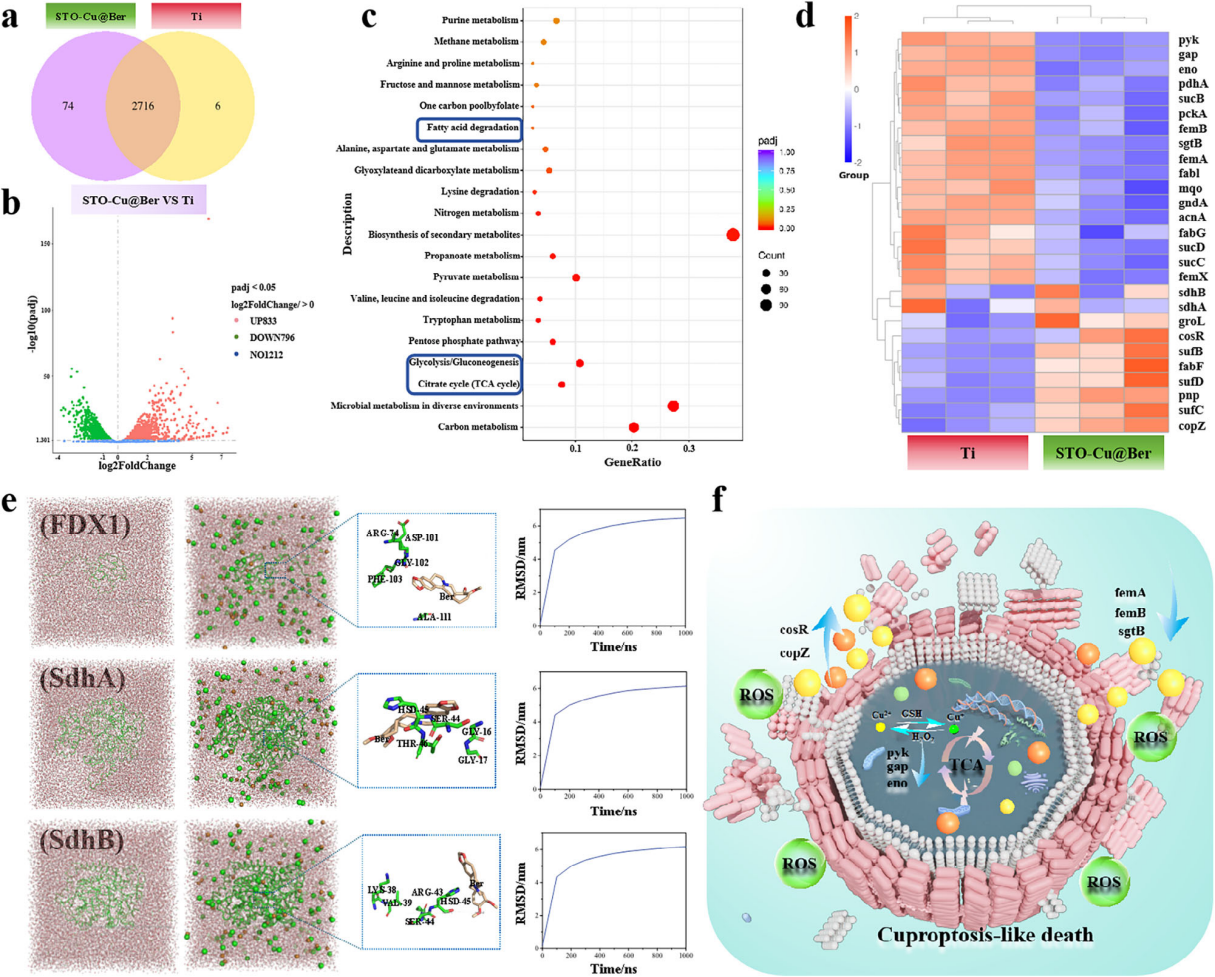
a) Venn diagram and b) Volcano plots of differentially expressed genes; c) KEGG enrichment of downregulated pathways; d) Heat map of differentially express genes; e) The snapshot of the STO‐Cu@Ber‐protein system during MD simulation; f) Schematic diagram of bacterial death mechanism.

A total RNA transcriptome sequencing of MRSA was used to explore the antibacterial mechanism of STO‐Cu@Ber. A total of 2716 genes were found to be co‐expressed in both the Ti group and the STO‐Cu@Ber group, while 6 and 74 genes were uniquely expressed in the Ti and STO‐Cu@Ber groups, respectively (**Figure** **4**a). The gene expression volcano plot in Figure 4b illustrates that, compared with the Ti group, the expression of 833 genes in the STO‐Cu@Ber group was upregulated and 796 genes were downregulated under US irradiation. A comparative KEGG pathway analysis of differentially expressed genes (DEGs) between the Ti and STO‐Cu@Ber groups was performed (Figure 4c). Downregulated DEGs showed predominant enrichment in metabolic pathways, including fatty acid degradation, glycolysis/gluconeogenesis, and TCA cycle. Conversely, upregulated DEGs were primarily associated with nucleotide metabolism, base excision repair mechanisms, and fatty acid biosynthesis pathways.

Previous studies have demonstrated that cuproptosis‐like death depends on mitochondrial respiration, with cells relying on aerobic respiration exhibiting up to a thousand‐fold greater sensitivity to copper toxicity compared to those relying on glycolysis.^[^ ^4^ ^]^ Furthermore, excessive accumulation of Cu ions facilitates direct binding to TCA cycle‐associated thiolated components. This interaction induces the aggregation of lipoylated proteins and the concurrent depletion of iron–sulfur (Fe─S) cluster proteins.^[^ ^9c^ ^]^ These findings prompted further investigation into whether cuproptosis‐like death occurs in bacteria.

As shown in Figure 4d, fatty acids and peptidoglycan are crucial components of the bacterial cell membrane and wall, respectively.^[^ ^29^ ^]^ The downregulation of genes involved in fatty acid and peptidoglycan synthesis and degradation (e.g., *femA*, *femB*, *sgtB*) suggests damage to both the bacterial cell membrane and wall. This observation is consistent with previous TEM findings and with experimental results involving ONPG and protein leakage. The increased expression of the copper‐sensing transcriptional repressor (*cosR*) and copper chaperone (*copZ*) indicates a disruption in the bacterial copper transport system.^[^ ^8^ ^]^ Consequently, damage to the bacterial cell envelope facilitates rapid Cu ion influx. The downregulation of glycolysis‐related genes (*pyk*, *gap*, *eno*) implies that intracellular copper is reduced to the more toxic Cu⁺ state, which induces a shift in bacterial respiration and increases sensitivity to cuproptosis‐like death. Changes in the expression of TCA cycle‐related genes further support the occurrence of cuproptosis‐like death in bacterial cells. The combination of Cu and lipoylation enzymes results in the accumulation of lipoylated proteins.^[^ ^6^ ^]^ Gene expression changes (e.g., *pdhA*, *sucC*, *sucD*) indicate that STO‐Cu@Ber influences lipoylated protein formation under US irradiation. Additionally, Fe─S clusters serve as essential cofactors for core TCA cycle enzymes. Dysregulation of genes such as *sdhA*, *sdhB*, *sufC*, and *sufD* compromises electron transport enzyme functionality, thereby disrupting TCA cycle continuity (Figure S13, Supporting Information). The accumulation of PDHA and the depletion of SDHB observed in the Western blot (Figure S14, Supporting Information) further confirm the occurrence of cuproptosis‐like death rather than mere oxidative damage.

To further examine the effect of STO‐Cu@Ber on fatty acylated and Fe─S cluster proteins, all‐atom molecular dynamics simulations were conducted to assess interaction modes between STO‐Cu@Ber and relevant transferase homologues. As shown in Figure 4e, snapshots at t = 0 and 500 ns demonstrate that STO‐Cu@Ber significantly alters the conformations of lipoylation enzymes (FDX1) and Fe─S cluster synthase homologues (SDHA, SDHB). STO‐Cu@Ber modifies the conformation of these enzyme homologues by interacting with specific amino acid residues. The RMSD curve shows that the RMSD values for STO‐Cu@Ber increased to nearly 6 nm within 500 ns, suggesting substantial conformational changes. After 500 ns of simulation, the RMSD values stabilized, indicating that the complex structure observed at 500 ns reflects a stable conformation following interaction with the relevant enzyme homologues.

Based on this analysis, we propose a mechanism by which STO‐Cu@Ber eliminates MRSA under US irradiation. Initially, STO‐Cu@Ber generates a substantial amount of ROS and releases Cu ions and Ber upon US irradiation, compromising the integrity of the bacterial cell wall and membrane. This structural disruption facilitates enhanced intracellular accumulation of Cu ions and Ber. Subsequently, Cu ions are reduced to the more toxic Cu⁺ state, altering bacterial respiration. The accumulation of Cu ions disrupts the TCA cycle and causes substantial nutrient leakage, thereby accelerating bacterial death.

### 2.5 Anti‐Osteomyelitis In Vivo and Immune Response

**Figure 5:**
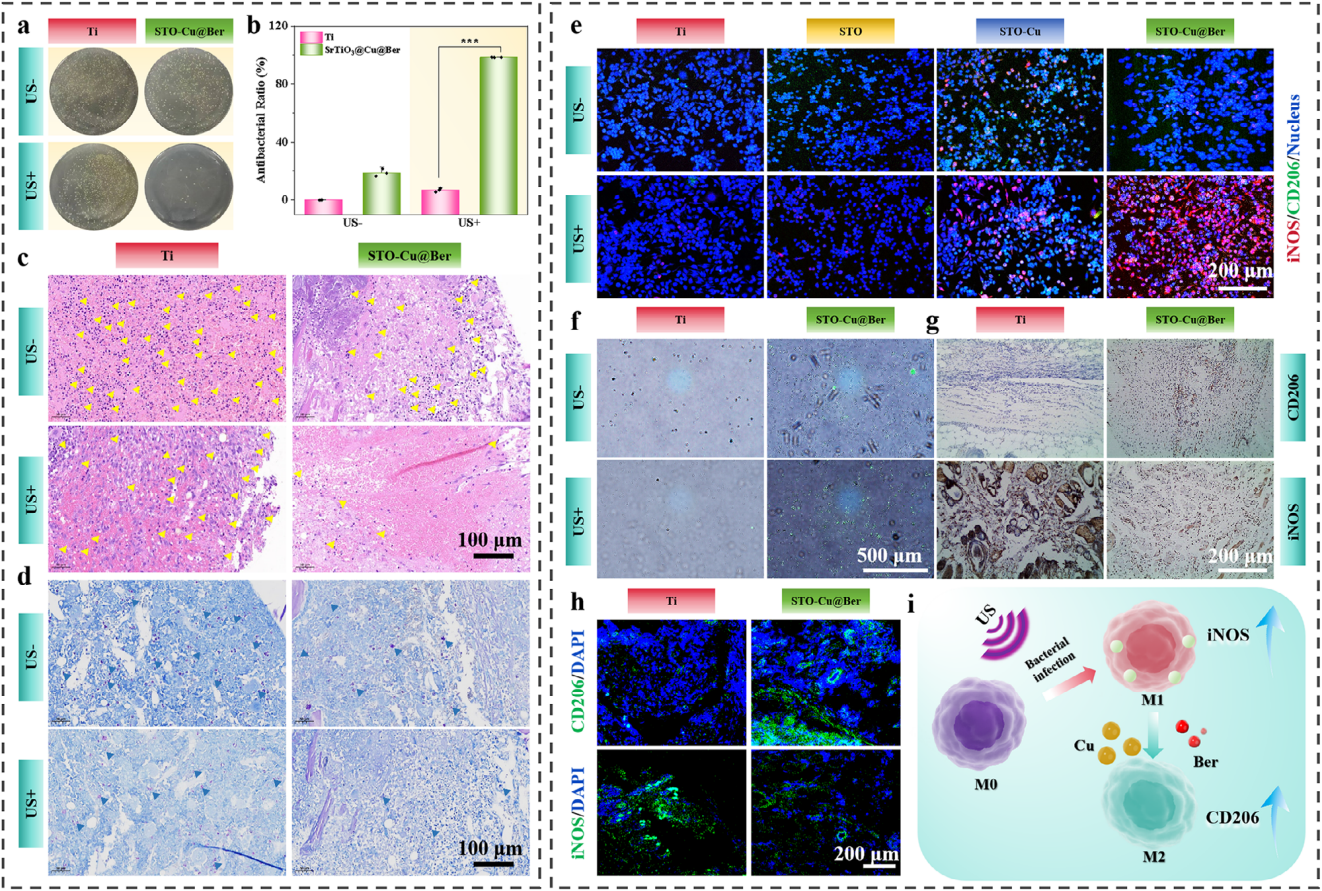
a) Bacterial colonies on Ti and STO‐Cu@Ber after different treatments (US‐ and US+) in vivo; b) Antibacterial rates (n = 3); c) H&E and d) Giemsa staining of peri‐implant bone tissues; e) Immunofluorescence images of RAW246.7; f) Images of bacterial phagocytosis by macrophages; g) Immunohistochemical staining and h) Immunofluorescence staining images of CD206 and iNOS in vivo; i) Schematic diagram of immunomodulation. Error bars represent ± SD. ***p < 0.001 by Student's *t*‐test.

The antibacterial effect of STO‐Cu@Ber in vivo under US irradiation was assessed using an osteomyelitis model of *S. aureus* infection. After two days of treatment, the remaining bacteria were collected for plate coating experiments, with the results shown in **Figure** **5**a,b. Notably, only the STO‐Cu@Ber group exhibited rapid bacterial killing following US irradiation, with an antibacterial rate as high as 98.4%. Osteomyelitis caused by bacterial infection leads to the rapid accumulation of inflammatory cells, such as neutrophils, at the infection site to initiate an immune response. However, excessive inflammation can ultimately result in implant failure. To evaluate the inflammatory response and bacterial load in the tissue surrounding the implant, H&E and Giemsa staining were performed. As shown in Figure 5c,d, only the STO‐Cu@Ber group exhibited reduced infiltration of inflammatory cells and fewer residual bacteria after US irradiation. These findings indicate that STO‐Cu@Ber is effective in rapidly eliminating bacteria in vivo and reducing inflammation.

Macrophages are pivotal mediators of immune homeostasis, and their functional plasticity directly modulates innate and adaptive host defense mechanisms. To further investigate the immunomodulatory effect of STO‐Cu@Ber under US irradiation, immunofluorescence was used to assess its influence on macrophage polarization. As shown in Figures 5e and S15 (Supporting Information), iNOS serves as a marker for M1 macrophages, while CD206 indicates M2 macrophages. The results demonstrate that STO‐Cu@Ber rapidly and markedly promotes macrophage polarization toward the pro‐inflammatory M1 phenotype under US irradiation. In addition, macrophage phagocytic activity against bacteria was confirmed through co‐culture experiments with bacteria and macrophages (Figure 5f). Under US irradiation, macrophages in the STO‐Cu@Ber group engulfed significantly more bacteria (indicated by green fluorescent dots) than those in other groups, suggesting that STO‐Cu@Ber enhances M1 polarization to increase macrophage phagocytic capacity. However, two days after US irradiation, immunohistochemical and immunofluorescence analyses revealed that the STO‐Cu@Ber group exhibited markedly higher CD206 expression and significantly lower iNOS expression (Figure 5g,h). These results suggest that STO‐Cu@Ber effectively resolves bacterial infection and suppresses inflammation after two days of US treatment, potentially due to the anti‐inflammatory activity of Ber released from the coating. As shown in Figure S16 (Supporting Information), flow cytometry indicated that macrophages in the STO‐Cu@Ber group showed a stronger M2 polarization trend compared to Ti, supporting the ability of STO‐Cu@Ber to resolve inflammation effectively. Previous studies have shown that Ber attenuates inflammation through three primary mechanisms: inhibition of inflammatory factor production, suppression of inflammatory cell infiltration and migration, and antioxidant effects.^[^ ^33^ ^]^ Furthermore, research has also demonstrated that implant surface nanostructures can promote macrophage polarization toward the M2 phenotype.^[^ ^34^ ^]^

In summary, we propose an immune regulatory mechanism for STO‐Cu@Ber (Figure 5i). In the early phase of bacterial infection, ROS generated by SPT induces macrophage polarization toward the M1 phenotype, facilitating rapid and cooperative bacterial clearance. Subsequently, the nanostructure of the coating and the intrinsic anti‐inflammatory activity of Ber promote macrophage polarization toward the M2 phenotype, accelerating the resolution of inflammation following bacterial clearance.

### 2.6 Metabolomics Analysis After IAOM Treatment

**Figure 6:**
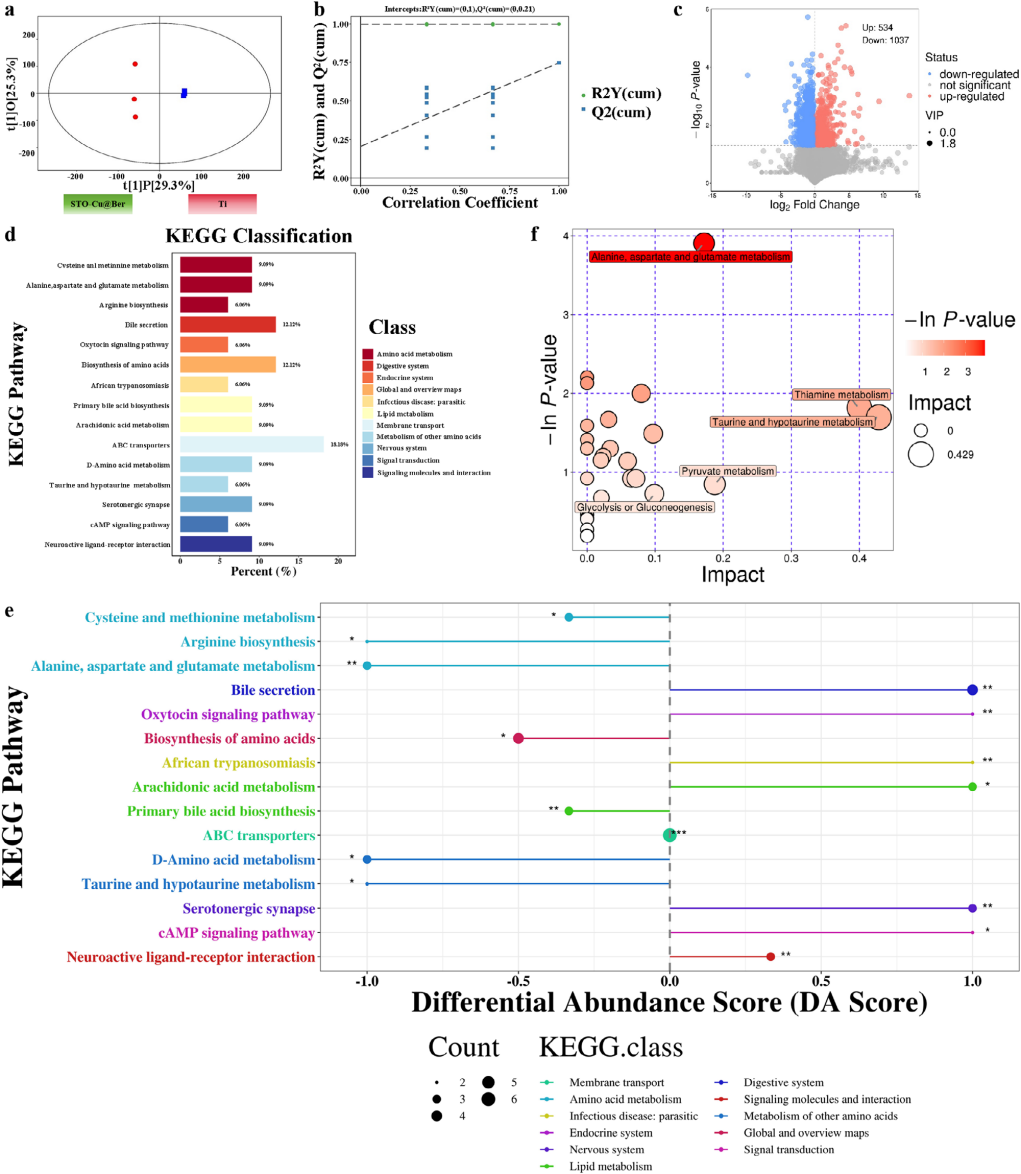
a) Multivariate score distribution in OPLS‐DA analysis (scatter visualization); b) Permutation validation plot of the OPLS‐DA model; c) Volcano plot for differential metabolite screening; d) Mapping of KEGG pathway classification; e) Visualization of differential Abundance Score; f) Characteristic bubble plot of KEGG pathways.

The role of STO‐Cu@Ber in the treatment of osteomyelitis was evaluated by analyzing metabolomic profiles following US treatment. As shown in the OPLS‐DA scatter and model plots (**Figure** **6**a,b), all sample data were valid and statistically significant. The volcano plot in Figure 6c indicates that, after US irradiation, the STO‐Cu@Ber group had 534 upregulated and 1037 downregulated metabolites compared with the Ti group.

Figure 6d presents the KEGG classification of differential metabolites. Amino acid metabolism and biosynthesis account for 45.45%, ABC transporters for 18.18%, arachidonic acid metabolism for 9.09%, and signal transduction‐related metabolites for 6.06%. These differences indicate that STO‐Cu@Ber significantly affects key biological processes such as energy production, immune response, and signal transduction under US irradiation.

The differential abundance score plot in Figure 6e shows the trends of specific metabolites in the STO‐Cu@Ber group following US irradiation. Compared with the Ti group, the downregulation of amino acids and biosynthesis‐related metabolites in the STO‐Cu@Ber group suggests a normalization of basal metabolism from the previously altered infection state. The downregulation of arachidonic acid metabolites and upregulation of the cAMP signaling pathway indicate suppression of the inflammatory response. Reduced levels of arachidonic acid metabolites result in decreased production of inflammatory mediators such as prostaglandin E_2_ (PGE_2_) and the chemokine leukotriene B_4_ (LTB_4_), thereby preventing the accumulation of inflammatory cells at the infection site.^[^ ^35^ ^]^ Simultaneously, this promotes the transformation of macrophages into an anti‐inflammatory phenotype (M2), reducing the secretion of inflammatory mediators such as tumor necrosis factor‐α (TNF‐α) and interleukin‐1β (IL‐1β), and effectively attenuating the inflammatory response.^[^ ^35b^ ^]^ cAMP serves as a critical intracellular second messenger, and elevated levels activate protein kinase A (PKA).^[^ ^36^ ^]^ Upon activation, PKA phosphorylates and inhibits the activity of nuclear factor‐κB (NF‐κB), a key transcription factor in the inflammatory response. NF‐κB drives the expression of inflammatory mediators, including IL‐1β and TNF‐α.^[^ ^37^ ^]^ Thus, inhibition of NF‐κB leads to decreased production of these mediators, contributing to the resolution of inflammation. Additionally, elevated cAMP levels suppress the pro‐inflammatory phenotype of macrophages, promoting their transition to the anti‐inflammatory M2 phenotype, as reflected by reduced release of pro‐inflammatory cytokines and increased production of anti‐inflammatory cytokines such as interleukin‐10 (IL‐10).^[^ ^38^ ^]^ Moreover, in bone tissue, increased cAMP pathway activity can enhance osteoblast differentiation and inhibit the bone‐resorptive activity of osteoclasts,^[^ ^39^ ^]^ which is essential for promoting bone integration after infection resolution.

As shown in Figure 6f, KEGG pathway analysis of enriched metabolites revealed involvement in amino acid metabolism, oxidative stress, and energy‐related processes. In rats with osteomyelitis, implantation of STO‐Cu@Ber followed by US irradiation led to consistent downregulation of these pathways, suggesting that the energy imbalance caused by bacterial infection was restored and the tissue matrix gradually returned to normal. In conclusion, STO‐Cu@Ber effectively eliminates osteomyelitis under US irradiation by suppressing inflammation and facilitating the rapid recovery of the tissue matrix.

### 2.7 Osseointegration Capacity of STO‐Cu@Ber

**Figure 7:**
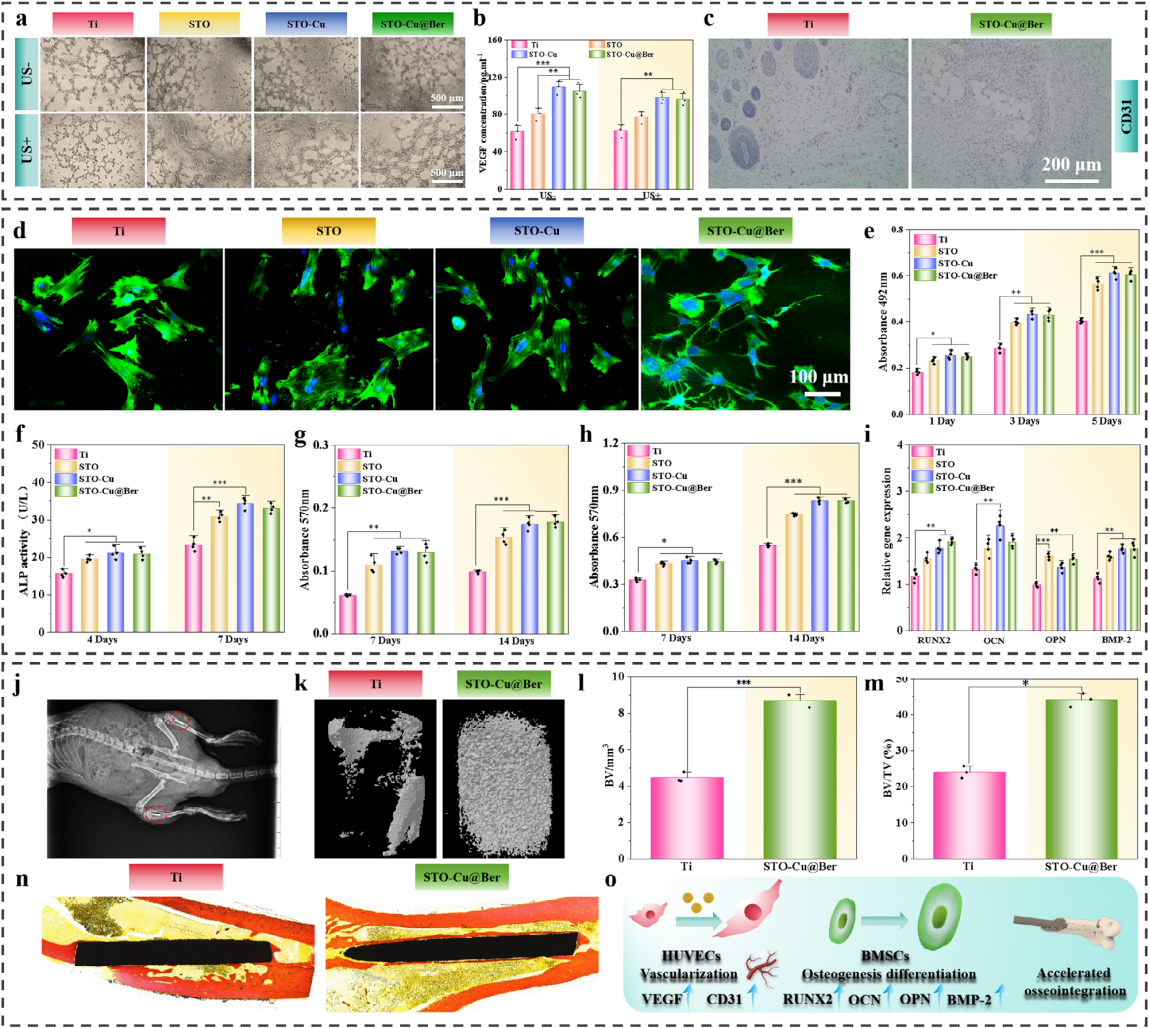
a) Angiogenesis ability of HUVECs; b) VEGF concentrations secreted by HUVECs (n = 3); c) Immunohistochemical staining images of CD31 in vivo; d) Skeleton assembly and e) MTT results of BMSCs (n = 4); f) Quantitative analysis of ALP activity (n = 4), g) Collagen secretion (n = 4) and h) ECM mineralization of BMSCs (n = 4); i) The expression levels of osteogenic genes (n = 4); j) CT image of the rat tibia after implantation of the implant; k) 3D micro‐CT reconstruction of the tissue surrounding the implant; l) Quantitative analysis of BV (n = 3) and m) BV/TV (n = 3); n) Van Gieson's picro fuchsin staining of the tissue; o) Schematic diagram of STO‐Cu@Ber promoting osseointegration. Error bars represent ± SD. *p < 0.05, **p < 0.01, ***p < 0.001 by Student's *t*‐test.

Angiogenesis plays a crucial role in osseointegration at the bone‐implant interface, as it facilitates integration through various mechanisms and is influenced by multiple factors.^[^ ^23^ ^]^ Therefore, the effect of STO‐Cu@Ber under US irradiation on human umbilical vein endothelial cells (HUVECs) was evaluated. The angiogenic capacity of the cells on the sample surfaces under different treatments (US− and US+) after two days was assessed using an angiogenesis kit containing ECMatrix gel, evaluated at 12 h. As shown in **Figure** **7**a, US irradiation had little effect on the angiogenic capacity of the cells on each group of samples. Although SPT may cause minor cellular damage, the cells can self‐repair within a short period and return to a normal state. In addition, the number of nodes and circles and the formation of tubular structures in the STO, STO‐Cu, and STO‐Cu@Ber groups were significantly higher than in the Ti group, indicating their potential to promote angiogenesis. VEGF is a key regulator of vascular morphogenesis. The ELISA method was used to quantitatively determine VEGF levels as an indicator of angiogenic potential. Similarly, the presence or absence of US irradiation had little effect on VEGF expression. VEGF levels increased in the STO group compared to the Ti group and further increased in the STO‐Cu and STO‐Cu@Ber groups, with little difference between the latter two. Furthermore, CD31 immunohistochemical analysis of peri‐implant tissues in rats at the early stage revealed that angiogenesis in the STO‐Cu@Ber group was markedly enhanced compared to the Ti group (Figure 7c). This effect is likely due to the proangiogenic properties of Sr and Cu ions. Studies have shown that STO can enhance HUVEC proliferation and migration. Sr ions can bind to negatively charged proteins and glycosaminoglycans, which play key roles in angiogenesis.^[^ ^20^ ^]^ Sr ions also upregulate VEGF expression by activating intracellular signaling pathways such as the PI3K–Akt pathway.^[^ ^40^ ^]^ Similarly, Cu ions can activate signaling pathways related to VEGF expression. For example, Cu ions regulate the activity of protein kinase C (PKC),^[^ ^41^ ^]^ a key kinase in the VEGF pathway, whose activation triggers downstream phosphorylation events.^[^ ^42^ ^]^ Appropriate concentrations of Cu ions can also enhance the activity of endothelial nitric oxide synthase (eNOS), promoting nitric oxide (NO) production.^[^ ^43^ ^]^ NO stimulates HUVEC proliferation and migration, modulates vascular smooth muscle relaxation, and creates favorable hemodynamic conditions to support neovascular growth and maturation.^[^ ^23^ ^]^ As shown in Figure S17 (Supporting Information), compared with the control group, the STO‐Cu@Ber group significantly promoted HUVEC migration. The migration rate in the STO‐Cu@Ber + NO inhibitor group fell between that of the control and STO‐Cu@Ber groups. In contrast, the STO‐Cu@Ber + NO exogenous donor group significantly inhibited HUVEC migration. These results support the proposed Cu^2^⁺ → eNOS → NO pathway and illustrate how STO‐Cu@Ber maintains therapeutic NO levels via controlled Cu^2^⁺ release to avoid pathological effects, whereas excessive NO inhibits HUVEC viability.

Bone marrow mesenchymal stem cells (BMSCs) are also essential contributors to bone regeneration.^[^ ^29^ ^]^ Therefore, the effects of STO‐Cu@Ber on BMSCs were further investigate. Figure 7d shows fluorescence cytoskeleton staining of BMSCs on the sample surfaces. Compared with Ti, cells on the STO, STO‐Cu, and STO‐Cu@Ber surfaces exhibited greater spreading and more pseudopodia. This may be due to the combined effects of Sr ions and nanostructures in promoting BMSC adhesion, spreading, and differentiation. MTT results also indicate that STO, STO‐Cu, and STO‐Cu@Ber enhance BMSC proliferation compared with Ti. Notably, STO‐Cu and STO‐Cu@Ber exhibited the highest cell viability, possibly due to the controlled release of Cu ions, which further promoted proliferation.

The osteogenic differentiation of BMSCs was also evaluated. As shown in Figure 7f–h, at various time points, alkaline phosphatase (ALP) activity, collagen secretion, and extracellular matrix (ECM) mineralization of BMSCs on the STO, STO‐Cu, and STO‐Cu@Ber surfaces were higher than on Ti, with differences becoming more pronounced over time. Expression levels of osteogenic genes (*RUNX2*, *OCN*, *OPN*, *BMP‐2*) were assessed via real‐time quantitative polymerase chain reaction (RT‐qPCR). Similarly, cells on the STO, STO‐Cu, and STO‐Cu@Ber surfaces expressed these genes at higher levels than those on Ti

Previous studies have demonstrated that unique nanostructures on material surfaces can provide more attachment sites for cells, allowing them to better extend pseudopodia and form tight adhesion with the surface.^[^ ^29^ ^]^ Nanostructures can also regulate the expression of cell adhesion‐related proteins. These proteins further enhance the adhesion between cells and nanostructures, which may provide a stable basis for subsequent cellular activities such as differentiation and proliferation.^[^ ^44^ ^]^ Certain studies have demonstrated that Sr ions can stimulate osteoblast proliferation by activating relevant signaling pathways, including the mitogen‐activated protein kinase (MAPK) pathway.^[^ ^45^ ^]^ Sr ions are also capable of enhancing ALP activity through stimulation of signaling pathways such as the Wnt/β‐catenin pathway.^[^ ^46^ ^]^ Upon activation of the Wnt/β‐catenin pathway, β‐catenin accumulates in the cell and translocates to the nucleus, where it interacts with transcription factors of the TCF/LEF family to promote the transcription of osteogenesis‐related genes, including those encoding ALP.^[^ ^47^ ^]^ The presence of Sr ions can regulate the growth and deposition of calcium phosphate crystals, rendering the bone matrix mineralization process more orderly.^[^ ^47^ ^]^ In addition, an appropriate amount of Cu ions can regulate bone morphogenetic protein (BMP) and MAPK signaling pathways to promote the proliferation and osteogenic differentiation of BMSCs.^[^ ^48^ ^]^ The combined effects of these factors may jointly contribute to the promotion of BMSC proliferation and osteogenic differentiation by STO‐Cu@Ber.

Osteomyelitis can impede the osseointegration of implants. Therefore, new bone formation in vivo following osteomyelitis treatment with STO‐Cu@Ber was further investigated. Figure 7j validates the accuracy of the implant placement. After 8 weeks of implantation, micro‐CT scanning of 3D bone tissue surrounding the implants revealed significantly greater new bone formation around STO‐Cu@Ber compared with Ti (Figure 7k). Quantitative analysis of bone volume (BV) and bone volume/tissue volume (BV/TV) demonstrated that the values for STO‐Cu@Ber (8.72 and 44.6%, respectively) were significantly higher than those for Ti (4.51 and 24.4%, respectively) (Figure 7l,m). Van Gieson staining (Figure 7n) further confirmed that a substantial amount of newly formed bone had developed around the STO‐Cu@Ber implant. These in vivo results indicate that STO‐Cu@Ber can effectively eliminate osteomyelitis caused by bacterial infection under US irradiation and can promote new bone formation around the implant by enhancing angiogenesis and osteogenic differentiation.

Subsequently, H&E staining was performed on the major organs (heart, liver, spleen, lung, and kidney) of the rats to evaluate the biosafety of the implants. As shown in Figure S18 (Supporting Information), no obvious abnormalities were observed in any of the organs, demonstrating the excellent biosafety of STO‐Cu@Ber.

## 3 Conclusion

In summary, the Herbal‐Piezo‐HJ was successfully prepared on Ti implants for the treatment of osteomyelitis. The construction of Herbal‐Piezo‐HJ reduces the interface bandgap, induces charge imbalance, and enhances the efficiency of SPT. ROS and Ber jointly damage the bacterial cell wall and membrane, increasing Cu ion influx into bacterial cells. Subsequently, the respiratory mode of bacterial glycolysis is altered, the TCA cycle is disrupted, and bacterial cells ultimately undergo cuproptosis‐like death. In addition, Herbal‐Piezo‐HJ can alleviate the immune response following osteomyelitis infection, promote angiogenesis and osteogenic differentiation, and enhance bone integration. The combination of herbal medicines and SPT improves the efficiency of inducing bacterial cuproptosis‐like death by trace amounts of Cu ions, providing a novel strategy for the treatment of IAOM.

## 4 Experimental Section

### Preparation of STO‐Cu@Ber

Medical‐grade Ti foils were sectioned into samples with dimensions of 1 cm × 1 cm × 0.2 mm, followed by sequential US cleaning in acetone, ethanol, and deionized water (labeled as Ti). The Ti substrates were immersed in an aqueous NaOH solution (1 mol L^−1^) and subjected to hydrothermal treatment at 220 °C for 4 h. After removal, the samples were ultrasonically rinsed in deionized water for 30 s and subsequently immersed in 0.5 mol L^−1^ HCl for 30 min. The acid‐treated samples were transferred to a 40 mL aqueous solution containing 6.67 mg of Sr(OH)_2_·8H_2_O and hydrothermally processed at 180 °C for 10 h (labeled as STO). The STO samples were then immersed in a 0.1 mol L^−1^ CuCl_2_ solution for hydrothermal treatment at 180 °C for 6 h. Post‐treatment, the samples underwent 1 min of US cleaning in deionized water, air‐dried, and annealed in a tube furnace at 420 °C for 1.5 h (labeled STO‐Cu). Finally, the STO‐Cu samples were immersed in a Ber solution (2 mg mL^−1^ in methanol) for 24 h. After rinsing and drying, the samples were labeled as STO‐Cu@Ber‌.

### Characterization

The FE‐SEM (JSM‐7001F, JEOL, Japan) was used to observe the surface morphology. Lattice fringes and elemental composition were analyzed using TEM (Tecnai G20, FEI, USA) equipped with EDS. The crystalline structure was identified via XRD (Rigaku Dmax‐3C, Cu Kα radiation), and surface elemental composition was analyzed by XPS (K‐Alpha, Thermo). Surface wettability was assessed by measuring contact angles using a goniometer (JC2000D2, Powereach, China). EPR spectra were recorded using an EPR spectrometer (EMXPLUS10/12, Bruker, Germany). Electrochemical properties were evaluated using an electrochemical workstation (CHI660E, Chenhua, China).

### Sonodynamic Properties

The sonodynamic performance of the samples was assessed by degradation experiments of MV and NBT and via EPR spectroscopy.^[^ ^49^ ^]^

### Ber Content Test

Ber content was quantified using UV–vis spectroscopy. The samples were ultrasonicated in 50% ethanol, and absorbance was measured. The surface concentration of Ber was determined by comparison with a standard concentration curve. The release kinetics of Ber were evaluated using the same method.

### Theoretical Calculations

DFT calculations were performed using the Vienna Ab initio Simulation Package (VASP 6.1.2). The Perdew–Burke–Ernzerhof (PBE) functional was employed to model electron exchange–correlation interactions. A Monkhorst‐Pack k‐point mesh with a 1×1×0 configuration was applied for Brillouin zone integration. The plane‐wave basis set was defined with a cutoff energy of 500 eV, and the electronic energy convergence tolerance was set to 10^−5^ eV.

### In Vitro Antibacterial Activities

Antibacterial efficacy against MRSA was evaluated by suspending samples in 1 mL of bacterial suspension (10⁵ CFU mL^−1^) and exposing them to US irradiation (1.5 W cm^−^^2^, 1 MHz, 50% duty cycle) for 15 min. A control group without ultrasound treatment was included. Bacterial viability was assessed by the spread plate method, and anti‐biofilm activity was evaluated using a bacterial live/dead kit (AO/PI).^[^ ^49^ ^]^

The degradation of GSH was analyzed by ultrasonically treating bacteria soaked in GSH, followed by the addition of Tris‐HCl buffer and 5,5′‐dithiobis(2‐nitrobenzoic acid), with absorbance measured at 420 nm. Bacterial membrane permeability and protein leakage were measured using ONPG and BCA kits, respectively.^[^ ^23^, ^29^, ^30^, ^49^ ^]^

### Transcriptome Analysis

RNA was extracted from US‐treated and control bacterial samples and sequenced by Novogene (Beijing, China).

### Cell Culture

HUVECs and BMSCs (isolated from the tibiae of 3‐week‐old rats) were cultured in a humidified incubator (5% CO_2_) at 37 °C. Cells were maintained in DMEM/α‐MEM supplemented with sodium bicarbonate (2.2 g L^−1^), streptomycin (0.133 g L^−1^), penicillin (0.0667 g L^−1^), and 10% fetal bovine serum (FBS). The specific experimental procedures were consistent with those used in the previous work.^[^ ^23^, ^29^, ^30^, ^49^ ^]^

### VEGF Secretion of HUVECs

VEGF secretion in HUVECs was quantified via ELISA (Abcam, UK) using supernatants collected after 24 h of culture.

### Angiogenesis of HUVECs

ECMatrix (Millipore, ECM625) was used for in vitro angiogenesis assessment. After overnight thawing at 4 °C, the gel was mixed with dilution buffer and solidified in pre‐cooled 96‐well plates (50 µL well^−1^, 37 °C, 45 min). Following treatment (with or without US), HUVECs (2×10⁴ cells) and collected medium were added to the matrix and incubated at 37 °C for 12 h. Imaging was performed at each interval using fluorescence microscopy (4 random fields).

### Cell Cytoskeleton

Fluorescence images of BMSCs on sample surfaces were obtained after staining.^[^ ^49^ ^]^

### Proliferation of BMSCs

Cell proliferation was assessed using the MTT assay at different time points.^[^ ^49^ ^]^

### Osteogenic Differentiation of BMSCs

Osteogenic differentiation was evaluated by measuring alkaline phosphatase activity (Beyotime P0321 kit), collagen secretion (Sirius Red staining), and extracellular matrix mineralization (Alizarin Red S) at defined time points.^[^ ^49^ ^]^

### Macrophage Culture

RAW 264.7 cells (TIB‐71, ATCC, VA), a murine macrophage cell line, were used in this study. Cells were cultured in DMEM supplemented with 10% FBS, 1% penicillin–streptomycin, and 100 ng mL^−1^ lipopolysaccharide (LPS) for activation.

### Immunofluorescence Staining

Macrophage polarization was assessed using iNOS (M1) and CD206 (M2) markers. Cells (5×10⁴/mL) were seeded in 24‐well plates in standard medium for 24 h, then activated with LPS‐supplemented medium for 12 h. After PBS washing, samples were fixed with 4% paraformaldehyde (1 h), permeabilized with saponin (20 min, room temperature [RT]), and blocked using QuickBlock (1 h, RT). Primary antibodies (iNOS 1:1000, CD206 1:2000; Abcam) were incubated overnight at 4 °C (500 µL well^−1^), followed by secondary antibodies (Alexa Fluor 555/488, 300 µL well^−1^, 90 min, in the dark). Nuclei were stained with DAPI (5 min) and imaged using CLSM.

### MRSA/Macrophage Co‐Culture

MRSA suspension (1×10⁸ CFU mL^−1^) was fluorescently labeled with CFDA‐SE (20 min) for tracking. Labeled bacteria were combined with macrophages and subjected to experimental treatments. Co‐cultures were incubated on an orbital shaker (120 rpm, 37 °C, 3 h), followed by fluorescence imaging using CLSM.

### In Vivo Compliance

All animal experimental procedures were approved by the Animal Ethics Committee of Taiyuan University of Technology (TYUT‐202205001), and all procedures were conducted in accordance with specific ethical guidelines.

### Antibacterial Assessment

*S. aureus* suspension (50 µL, 1×10⁹ CFU mL^−1^) was injected into the tibia of rats to establish IAOM models (n = 18, 300–320 g, male SD rats). Rats were divided into Ti and STO‐Cu@Ber groups. Samples (Φ1.5 × 3 mm) were sterilized (UV, 10 min) and implanted into the tibias. Post‐surgery, wounds were irradiated with US (1.5 W cm^−^^2^, 1 MHz, 50% duty cycle) for 15 min. After 48 h, implants were retrieved for biofilm quantification. Peri‐implant tissues were fixed (4% PFA), embedded, sectioned, and stained (H&E/Giemsa) for histological analysis (3 random fields, Zeiss Imager M2). Immunostaining (iNOS/CD206/CD31) was performed to assess inflammation and angiogenesis.

### Metabolomics Analysis

Metabolomics testing was conducted by Biotree Biotech Co., Ltd. (Shanghai, China).

### New Bone Formation

New bone formation was analyzed in the remaining rats euthanized 30 days post‐surgery. Tibial segments containing the implants were fixed (4% PFA, 48 h), dehydrated, and scanned using micro‐CT (10 µm resolution) with defined thresholds (σ = 0.8, support = 1; bone = 35, implant = 211). Resin‐embedded samples were sectioned (50 µm) and stained with van Gieson's picro fuchsin for evaluation of osteogenesis.

### Statistical Analysis

Quantitative data are presented as mean ± standard deviation (SD) from at least three independent experiments (n ≥ 3). Statistical significance was determined using one‐way ANOVA followed by the Student‐Newman‐Keuls post hoc test in SPSS 14.0. Significance levels are indicated as *p < 0.05, **p < 0.01, and ***p < 0.001.

## Conflict of Interest

The authors declare no conflict of interest.

## Supporting information



Supporting Information

## Data Availability

The data that support the findings of this study are available from the corresponding author upon reasonable request.
