# Supplementary material for: A Herbal‐Piezoelectric Heterojunction Strategy to Potentiate Bacterial Cuproptosis‐Like Death and Remodel the Inflammatory Microenvironment in Infection‐Associated Implant Osteomyelitis
Source: Adv Sci (Weinh). 2025 Aug 28;12(43):e06362. doi: 10.1002/advs.202506362 (PMC12631926; doi:10.1002/advs.202506362)
Supplement: Supplementary file 1 — Supporting Information [file ADVS-12-e06362-s001.docx]

**Supplementary Information**

**Novel Herbal-Piezo-Heterojunction Strategy for Efficient Bacterial Cuproptosis-like Death and Inflammatory Microenvironment Remodeling in Infection-associated Implant Osteomyelitis**

Guannan Zhang ^#a,b^, Zehao Li ^#c^, Ying Lu ^a,b^, Jianbo Song *^a,b^, Xingyu Liang ^g^, Peide Han ^e^, Xiaohong Yao ^a^, Yongqiang Yang*^f^, Xiangyu Zhang *^c,d^

^a^ Shanxi Bethune Hospital, Shanxi Academy of Medical Sciences, Third Hospital of Shanxi Medical University, Tongji Shanxi Hospital, Taiyuan 030032, China

^b^ Shanxi Provincial Key Laboratory for Translational Nuclear Medicine and Precision Protection, Taiyuan 030006, China

^c^ Shanxi Key Laboratory of Biomedical Metal Materials, College of Materials Science and Engineering, Taiyuan University of Technology, Taiyuan 030024, China

^d^ College of Biomedical Engineering, Taiyuan University of Technology, Taiyuan 030024, China

^e^ College of Materials Science and Engineering, Taiyuan University of Technology, Taiyuan 030024, China

^f^ National Graphene Products Quality Inspection and Testing Center (Jiangsu), Special Equipment Safety Supervision Inspection Institute of Jiangsu Province, Wuxi 214174, China

^g^ The First Hospital of Shanxi Medical University, Taiyuan 030001, China

# These authors contributed equally

*Corresponding Author: E-mail: [zhangxiangyu@tyut.edu.cn](mailto:zhangxiangyu@tyut.edu.cn) (X Zhang)

*Corresponding Author: E-mail: [jianbo2611@s](mailto:jianbo2611@126.com)xmu.edu.cn (J Song)

*Corresponding Author: E-mail: yyqyyq_2008@163.com (Y Yang)


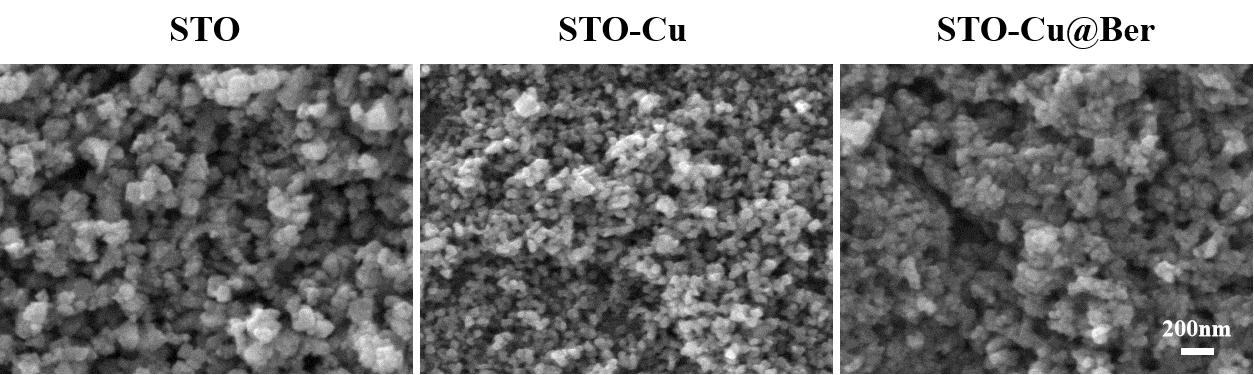


Figure S1. SEM images of STO, STO-Cu, STO-Cu@Ber.


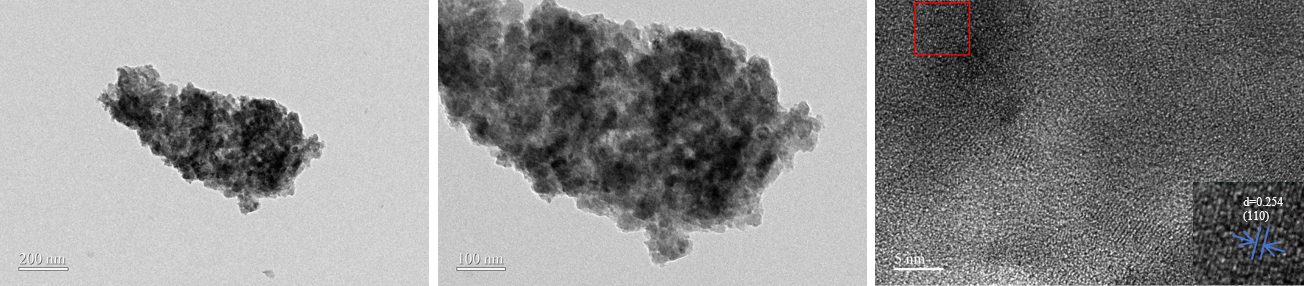


Figure S2. TEM images of STO.


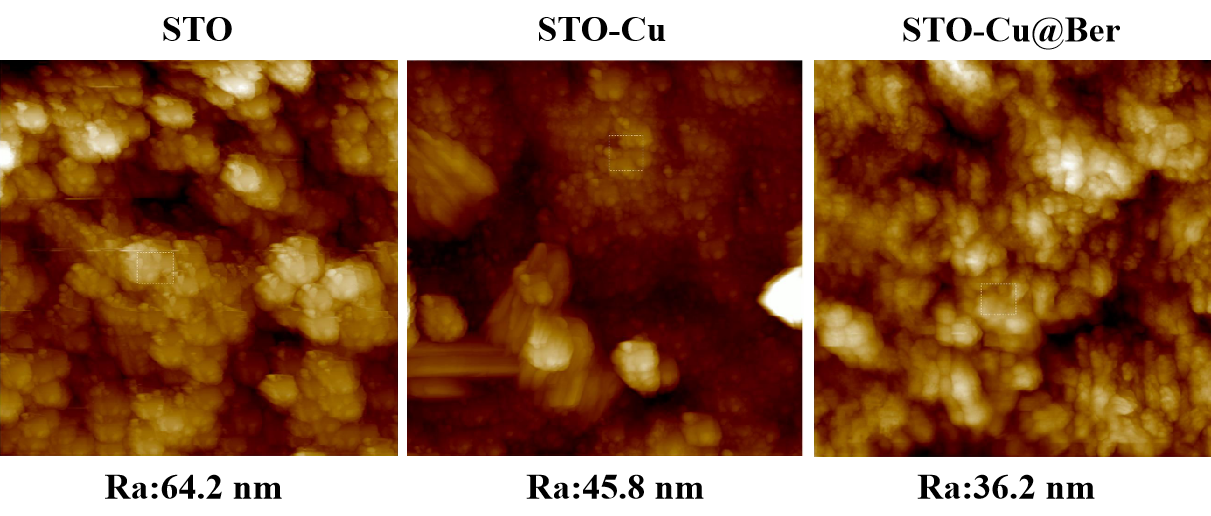


Figure S3. AFM images of STO, STO-Cu, STO-Cu@Ber.


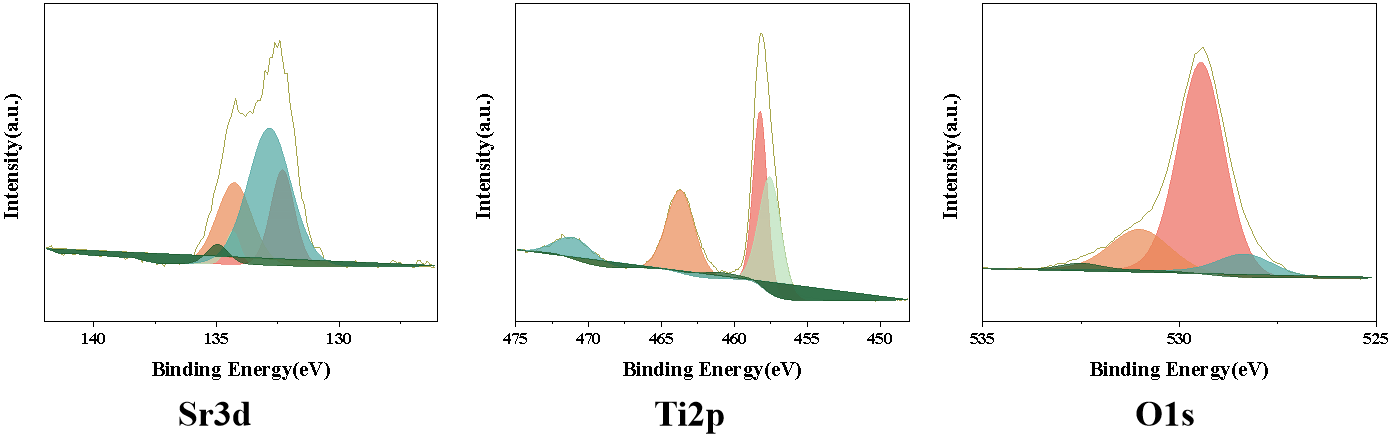


Figure S4. Sr3d,Ti2p and O1s high-resolution XPS spectra of STO.


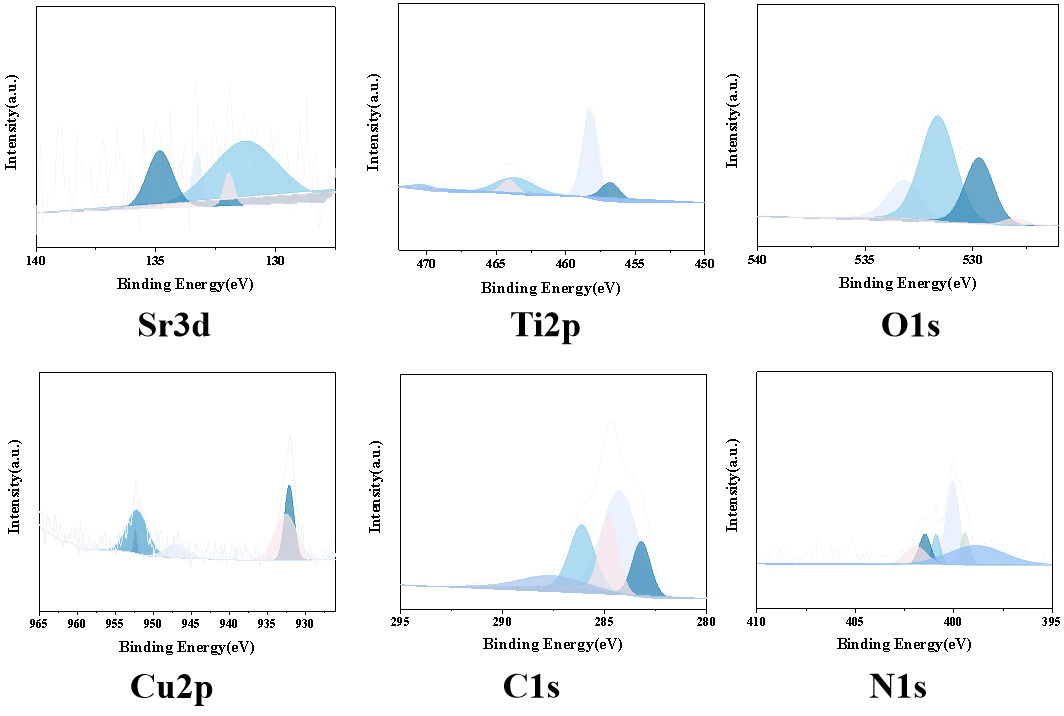


Figure S5. Sr3d,Ti2p, O1s, Cu2p, C1s and N1s high-resolution XPS spectra of STO-Cu@Ber.


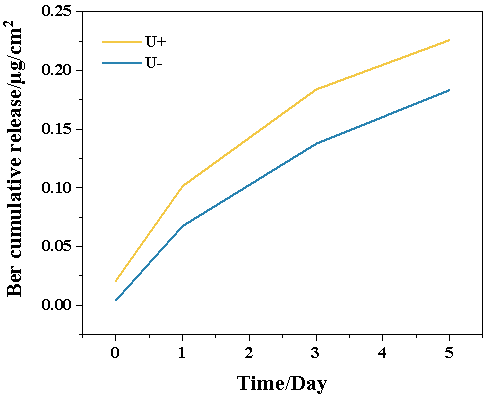


Figure S6. Kinetics of Ber release from STO-Cu@Ber surface.


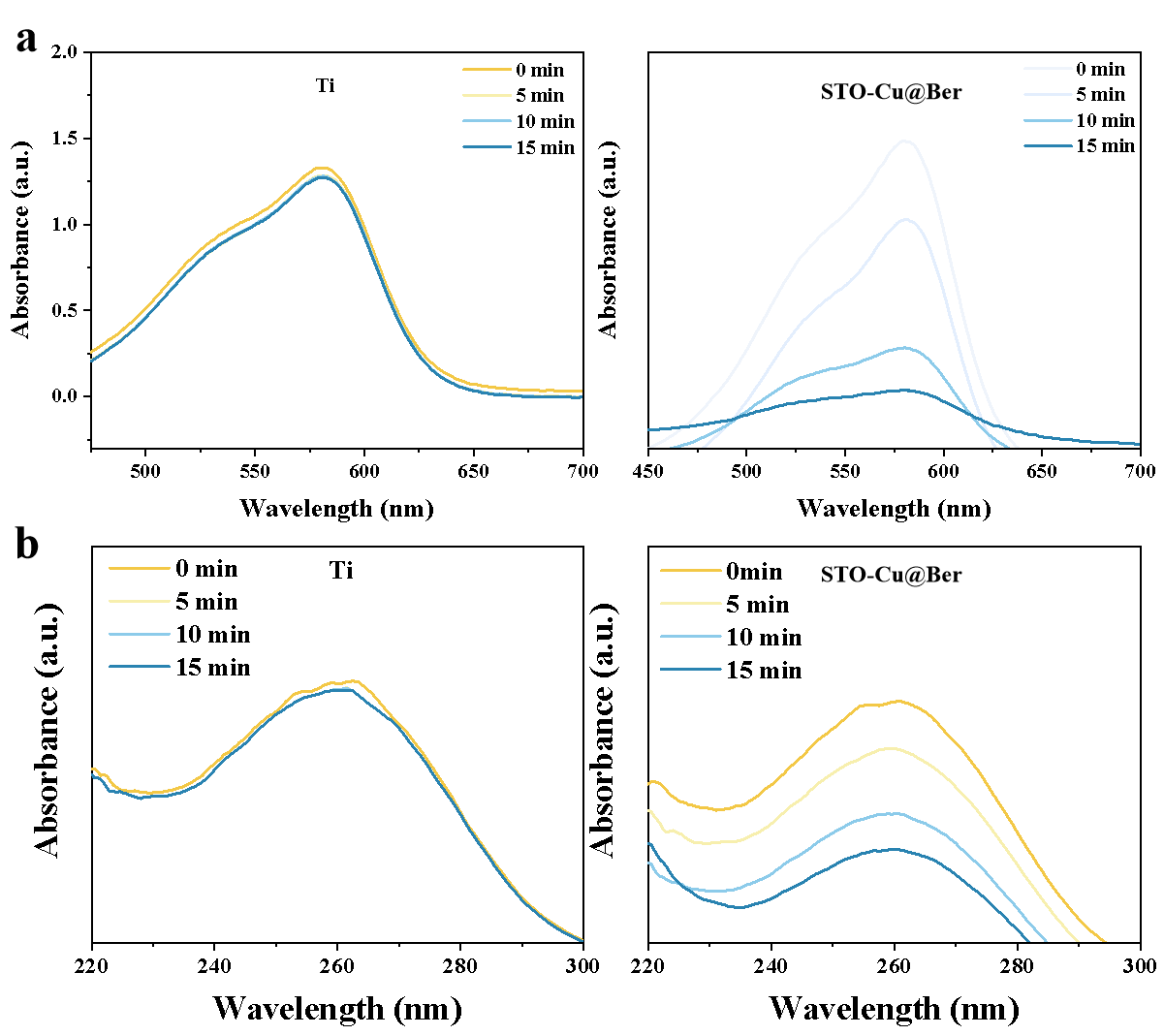


Figure S7. Degradation of MV and NBT at different times for Ti and STO-Cu@Ber.


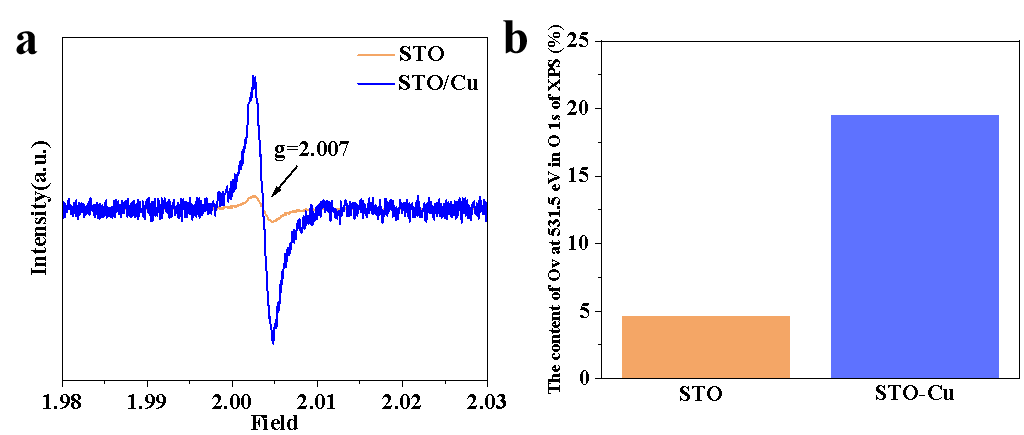


Figure S8. (a) Oxygen vacancy concentration in STO and STO-Cu detected by ESR spectroscopy and (b) XPS.


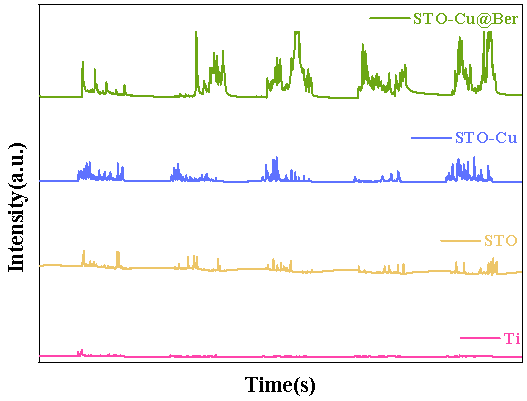


Figure S9. The ultrasonic current density of Ti, STO, STO-Cu, STO-Cu@Ber.


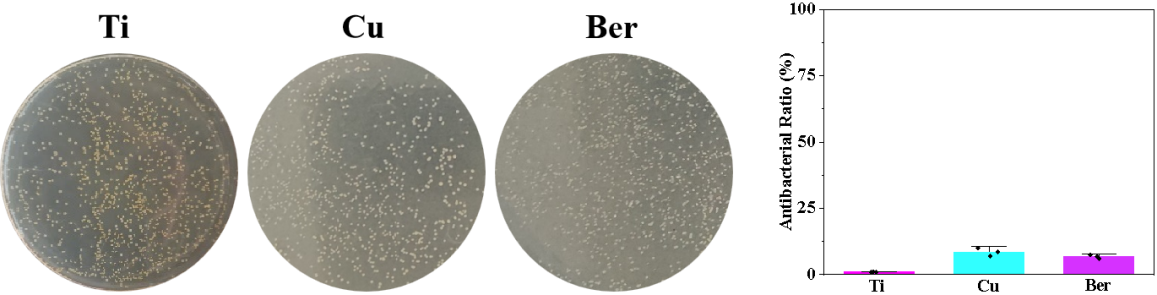


Figure S10. Antibacterial rates of Cu and Ber.

Figure S11. (a) GO enrichment analysis of gene functions of up-regulated and (b) down-regulated DEGs.

Figure S12. KEGG enrichment analysis of gene functions of up-regulated DEGs.


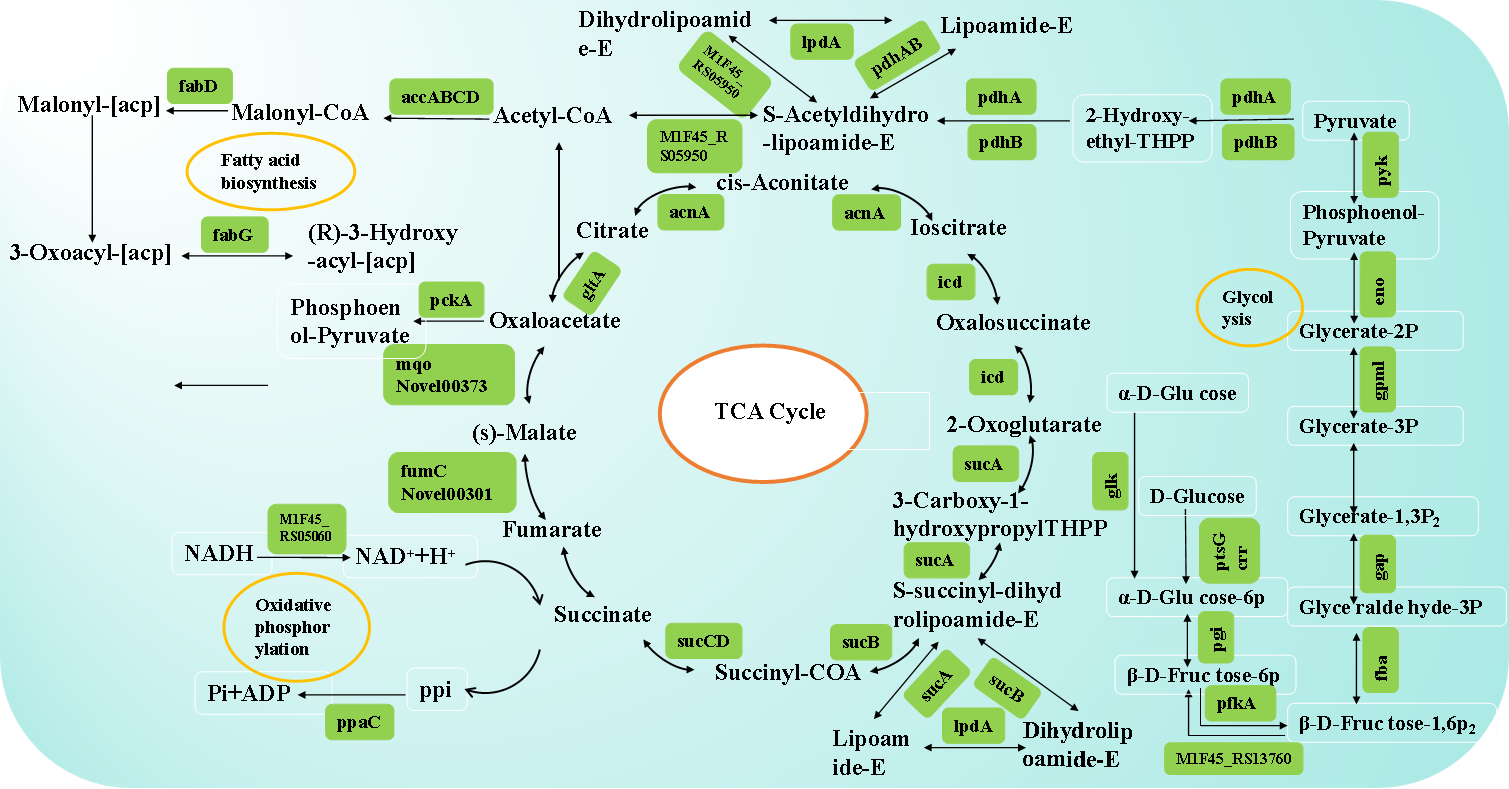


Figure S13. TCA cycle signaling pathway diagram.


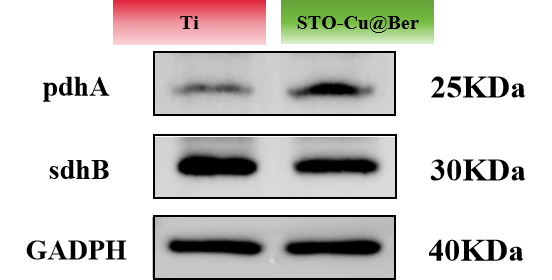


Figure S14. Western blot results of different protein expressions.


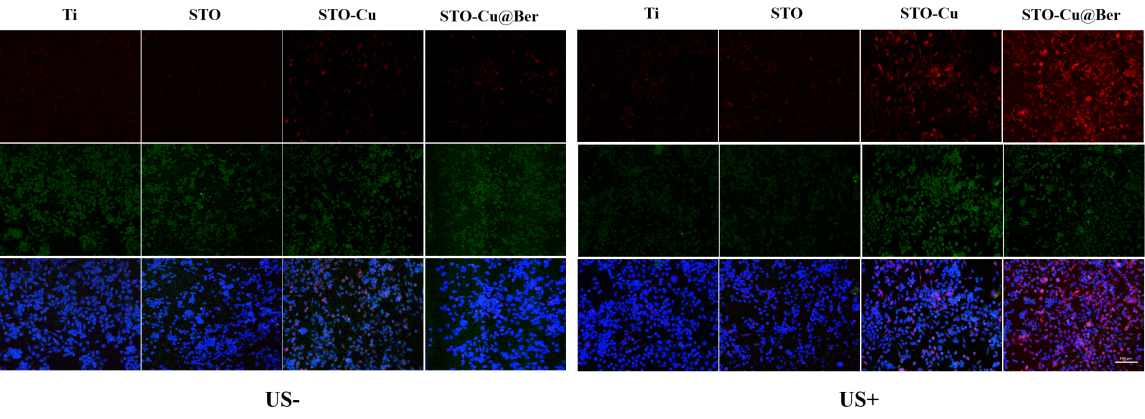


Figure S15. Immunofluorescence images of macrophage.


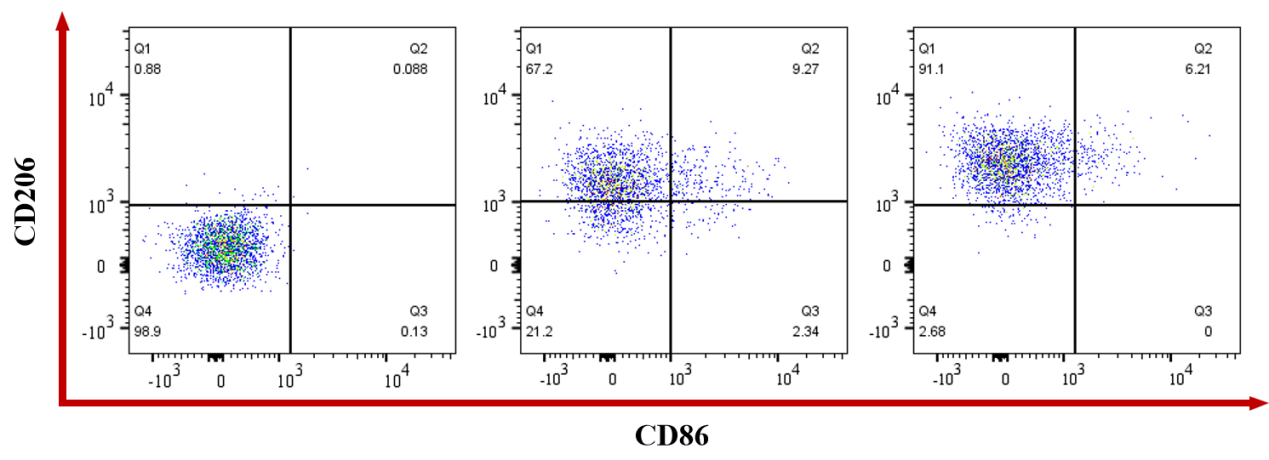


Figure S16. Flow cytometry results of Macrophage.


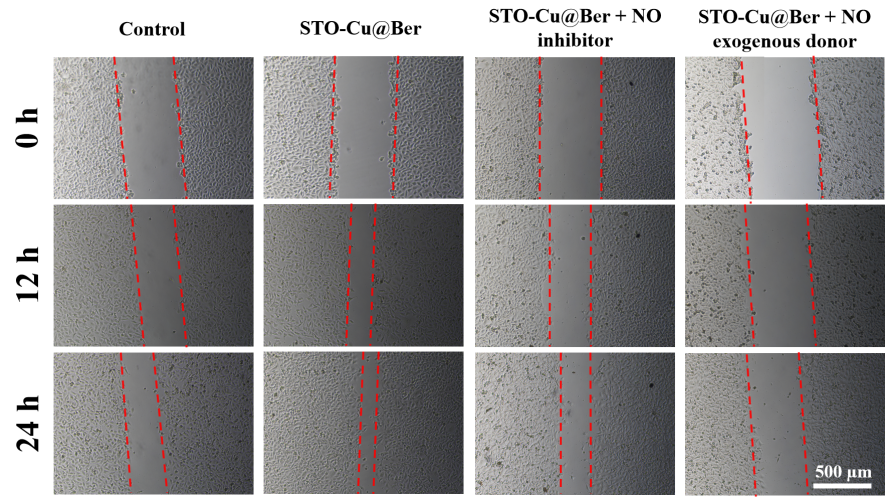


Figure S17. Images of the scratch assays of HUVECs.


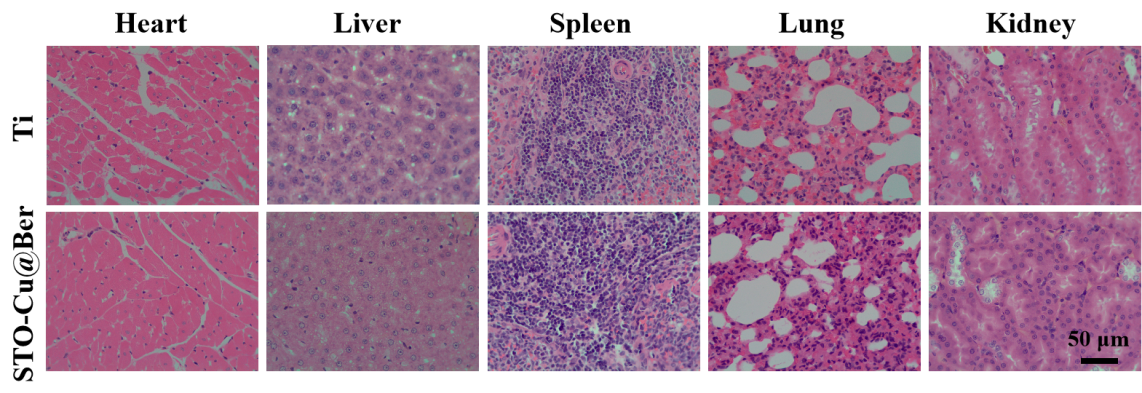


Figure S18. Histological analysis of major organs including the liver, spleen, kidney, heart and lung by H&E staining.

**Table S1.** ICP-OES test of STO-Cu@Ber.

| Element | Element concentration (mg/L) | Element content of sample (mg/kg) |
| --- | --- | --- |
| Ti | 666 | 1001536 |
| Sr | 0.0733 | 110.425 |
| Cu | 0.00447 | 6.7 |

**Table S2.** The elemental content of the bacteria in the STO-Cu@Ber group after US irradiation.

| Element | Atomic Fraction(%) | Atomic Error(%) | Mass Fraction(%) | Mass Error(%) | Fit error (%) |
| --- | --- | --- | --- | --- | --- |
| C | 81.78 | 1.78 | 59.83 | 2.83 | 1.72 |
| N | 1.33 | 0.27 | 1.14 | 0.24 | 4.45 |
| O | 9.09 | 1.67 | 8.86 | 1.67 | 1.65 |
| Cu | 7.8 | 1.05 | 30.18 | 3.09 | 0.08 |

**Table S3.** The primers for the target genes.

| Gene | Gene forward primer sequence (5’–3’) | Reverse primer sequence (5’–3’) |
| --- | --- | --- |
| GAPDH | GGTTGTCTCCTGCGACTTCA | TGGTCCAGGGTTTCTTACTCC |
| RUNX2 | AACCCACGAATGCACTACCCA | GGAACTGATAGGATGCTGACGAAG |
| OCN | AGACTCCGGCGCTACCTTGG | CGGTCTTCAAGCCATACTGGTCTG |
| OPN | ATCTCCTTGCGCCACAGAATGC | ATCTCCTTGCGCCACAGAATGC |
| BMP-2 | TGAACACAGCTGGTCTCAGG | ACCCCACATCACTGAAGTCC |
